# Polyarginine Peptide R11–Actin Interaction Induces a Domino Effect on Cytoskeleton Remodeling to Suppress Bladder Cancer Metastasis

**DOI:** 10.34133/research.1109

**Published:** 2026-01-29

**Authors:** Zhenghong Liu, Chuanzan Zhou, Wentao Xu, Dahong Zhang, Bin Zheng, Facai Zhang, Xiaowen Qin, Heng Wang, Yixuan Mou, Yang Liu, Haichang Li, Jing Quan, Li Sun, Yiyang Chen, Chenkai Wang, Xuanyi Zhou, Xinyi Chen, Hong Tang, Dingyi Liu, Wenyan Zuo, Dechao Feng, Pu Zhang, Qi Zhang

**Affiliations:** ^1^ Urology and Nephrology Center, Department of Urology, Zhejiang Provincial People’s Hospital, Affiliated People’s Hospital, Hangzhou Medical College, Hangzhou, Zhejiang 310014, China.; ^2^ State Key Laboratory of Advanced Technology for Materials Synthesis and Processing International School of Materials Science and Engineering Wuhan, University of Technology, Wuhan 430070, China.; ^3^ Department of Urology, The First Affiliated Hospital of Jinan University, Guangzhou, Guangdong 510630, China.; ^4^ Department of Nutrition and Food Hygiene, The National Key Discipline, School of Public Health, Harbin Medical University, Harbin 150081, China.; ^5^ Division of Surgery and Interventional Science, University College London, London W1W 7TS, UK.

## Abstract

Cytoskeletal remodeling, particularly actin dynamics, is a central driver of tumor metastasis. However, actin-targeting agents have faced major translational barriers due to poor specificity and the absence of defined druggable sites. Here, we report a bladder tumor-targeting polyarginine peptide, R11, as a precision modulator of actin dynamics capable of disrupting the cytoskeletal architecture of bladder cancer (BCa) to suppress its lung metastasis potently and persistently. R11 directly interacts with actin, weakening the actin–plectin–vimentin/integrin β4 axis and initiating a cascade of cytoskeletal disorganization that ultimately impairs cellular motility and metastatic potential. Remarkably, nanoscale multivalent assemblies of R11 amplify these effects through enhanced multivalent binding to actin. This study unveils a new strategy for cytoskeleton-targeted intervention through peptide-based precision materials, highlighting R11 assemblies as a promising therapeutic platform for the treatment of metastatic BCa and potentially other cytoskeleton-dependent malignancies.

## Introduction

Cytoskeletal remodeling is central to facilitating tumor metastasis. This intricate fiber network [1] is broadly defined into 3 components: microfilaments (MFs), intermediate filaments (IFs), and microtubules (MTs) [2]. The crosstalk among MFs, IFs, and MTs ensures their coordination in the remodeling process via master regulators [3], which additionally contribute to connecting the cytoskeleton to the extracellular matrix (ECM) [4] and regulating the cell–cell interface [5]. Therefore, multifaced communication that converges in the cytoskeleton can occur through mechanical–chemical signal coupling, thereby integrating all the signals together to jointly regulate tumor metastasis [6].

Actin is the primary component of MFs. The intracellular transition between monomer (G-actin) and assembly (F-actin) [7–9] is tightly and flexibly controlled to define cell behavior, particularly cell migration [3,10]. This biological process becomes more dynamic when tumor cells begin metastasis. Accordingly, actin is rapidly remodeled to enable adaptation to tumor metastasis [11]. Although bladder cancer (BCa) metastasis occurs through various pathways, such as lymph node metastasis mediated by vascular endothelial growth factor C (VEGFC)/vascular endothelial growth factor receptor 3 (VEGFR3), hematogenous spread, and direct invasion, we believe that the most relevant pathway remains actin within the cytoskeleton [12]. Many actin-targeting drugs have been developed but not translated to the clinic [13]. Small molecules, including phallodin, jasplakinolide, and latrunculin A, and other cationic polymers [7] and cationic peptides [14] lack uptake preference by specific types of cells, possibly leading to severe off-target toxicity [15]. Many actin regulators, as structural proteins, are also undruggable because of their lack of clear functional sites suitable for targeted drug design [16,17].

**Fig. 1.**
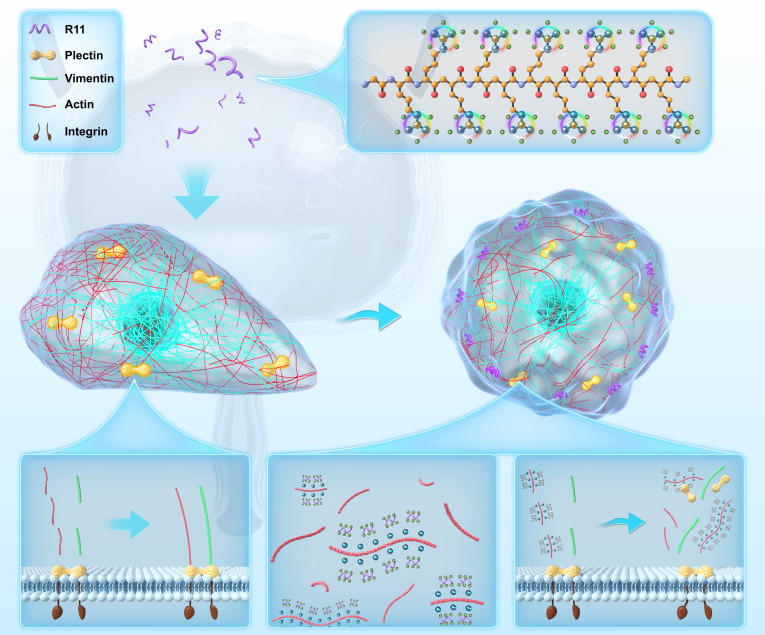
R11–actin interplay induces domino effect in cytoskeleton remodeling to inhibit BCa metastasis. R11 strongly interacted with actin to disrupt the stability of their tetramer. Therefore, the actin dynamics became unbalanced and the actin–plectin binding got diminished. Finally, vimentin/ITGβ4, originally together with plectin, fell apart, disorganizing the whole cytoskeleton and making BCa cells losing the polarity.

Leveraging the bladder tumor-targeting property and rapid transmembrane kinetics of the polyarginine peptide R11 [18], we employed R11 as a tumor-specific and actin-targeting drug to strongly and enduringly inhibit BCa lung metastasis. R11–actin interplay triggered the domino effect in cytoskeleton remodeling. Specifically, the insertion of the strongly positive R11 into the G-actin tetramer disrupted the stability of the tetramer and disrupted actin dynamics, subsequently diminishing actin–plectin binding. Finally, vimentin/integrin β4 (ITGβ4), which were originally together with plectin, fell apart, disorganizing the whole cytoskeleton (Fig. 1). Furthermore, the nanoscale R11 assemblies displayed enhanced actin-disrupting and antimetastatic abilities due to their multivalent reactions with actin.

## Results and Discussion

### Inhibitory effects of R11 on BCa metastasis

**Fig. 2.**
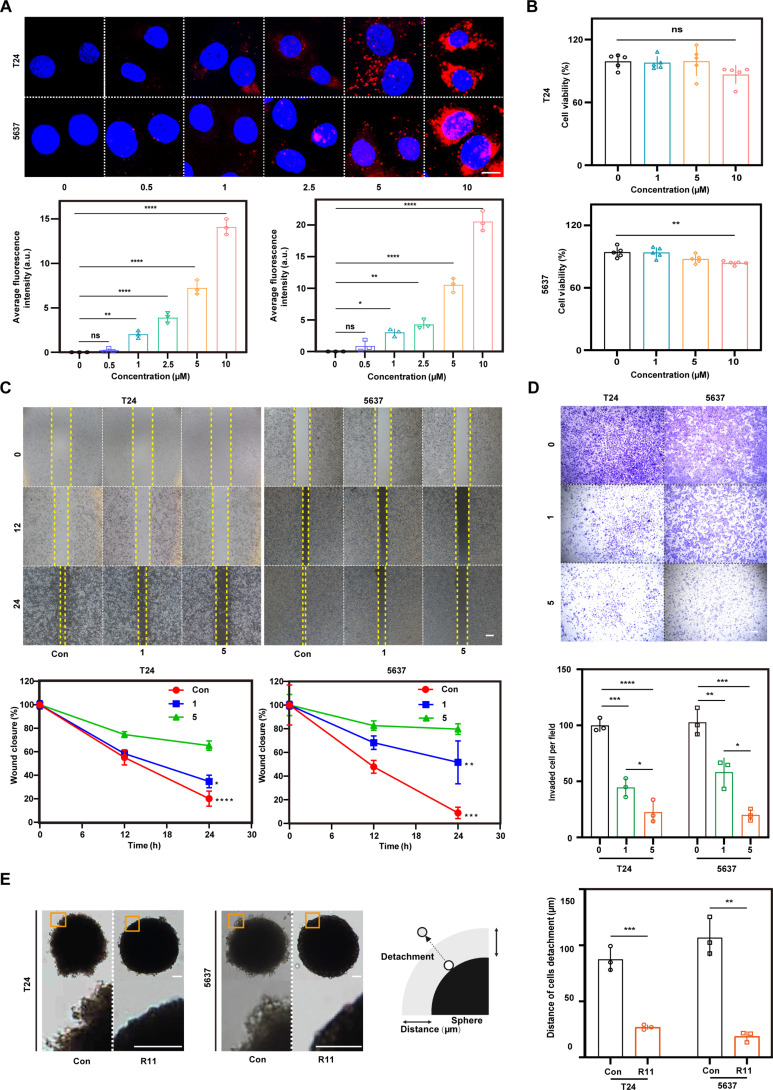
The anti-metastasis effect of R11 in vitro. (A) Cellular uptake of R11 in T24 and 5637 cells under different concentrations, observed via CLSM (upper insert). Nucleus was observed via DAPI fluorescence channel; R11 was observed via carboxytetramethylrhodamine (TAMRA) fluorescence channel; *n* = 3 independent experiments; scale bar: 10 μm. The average fluorescence of intracellular R11 (lower inserts). (B) Cytotoxicity of R11 of different concentrations in BCa cell lines; *n* = 5 independent experiments. (C) Migrating ability of T24 and 5637 cells, as determined by scratch wound healing assay (upper inserts); R11 (1 μM), 24-h incubation; R11 (5 μM), 24-h incubation. The average percentage of wound healing by the corresponding cells in upper inserts (lower inserts); *n* = 3 independent experiments; scale bar: 200 μm. (D) Bright-field imaging of cell migration, illustrated by the Transwell assay (upper inserts); R11 (1 M), 24-h incubation; R11 (5 μM), 24-h incubation; *n* = 3 independent experiments. The average number of transmembrane cells in the Transwell assay (lower inserts); scale bar: 200 μm. (E) Bright-field imaging of cell detachment from tumor spheroids (left inserts). The average thickness of outer detached layer (right inserts); R11 (5 μm), 24-h incubation; *n* = 3 independent experiments; scale bar: 50 μm. *P < 0.05; **P < 0.01; ***P < 0.001; ****P < 0.0001.

R11 is a cationic polyarginine peptide with the ability to rapidly penetrate cells and target BCa [18]. In this work, the cellular uptake of R11 was tested at different concentrations ranging from 0 to 10 μM. The internalization of R11 became more rapid as the concentration increased, and its subcellular distribution expanded to the nucleus once the concentration exceeded 1 μM (Fig. 2A). Its cytotoxicity profile in tumor cells demonstrated that 10 μM R11 decreased the viability of 5637 cells by 15%, and 5 μM R11 seemed to be a safe threshold for tumor cells bearing the R11 threat (Fig. 2B). A previous study offered very limited evidence on the therapeutic effect of R11 in treating tumors, as a common belief was that it is very weak. Surprisingly, we found that R11 impaired tumor metastasis at concentrations below the safe threshold. Wound healing assays revealed that after 12 h of incubation with 5 μM R11, 80% of the 5637 cells were inhibited from migrating to cover the wound. Similarly, in the Transwell migration assay, 24 h of incubation with R11 (5 μM) resulted in approximately 70% inhibition of transmembrane migration in both BCa cell lines (Fig. 2C and D). Multicellular spheroids (MCSs) represent an ideal 3-dimensional (3D) model to mimic tumors in vivo. The detachment of cells from MCSs better reflects a key biological process of tumor metastasis than monolayer cell culture models [19]. As depicted in Fig. 2E, in MCSs not subjected to R11 treatment, the cells grew out of the presumptive spheroid border and made the MCS border rough, whereas R11 strongly inhibited the formation of the outer detached layer, which was 50% less thick than that of the phosphate-buffered saline (PBS)-treated T24/5637 MCSs (Fig. 2E).

**Fig. 3.**
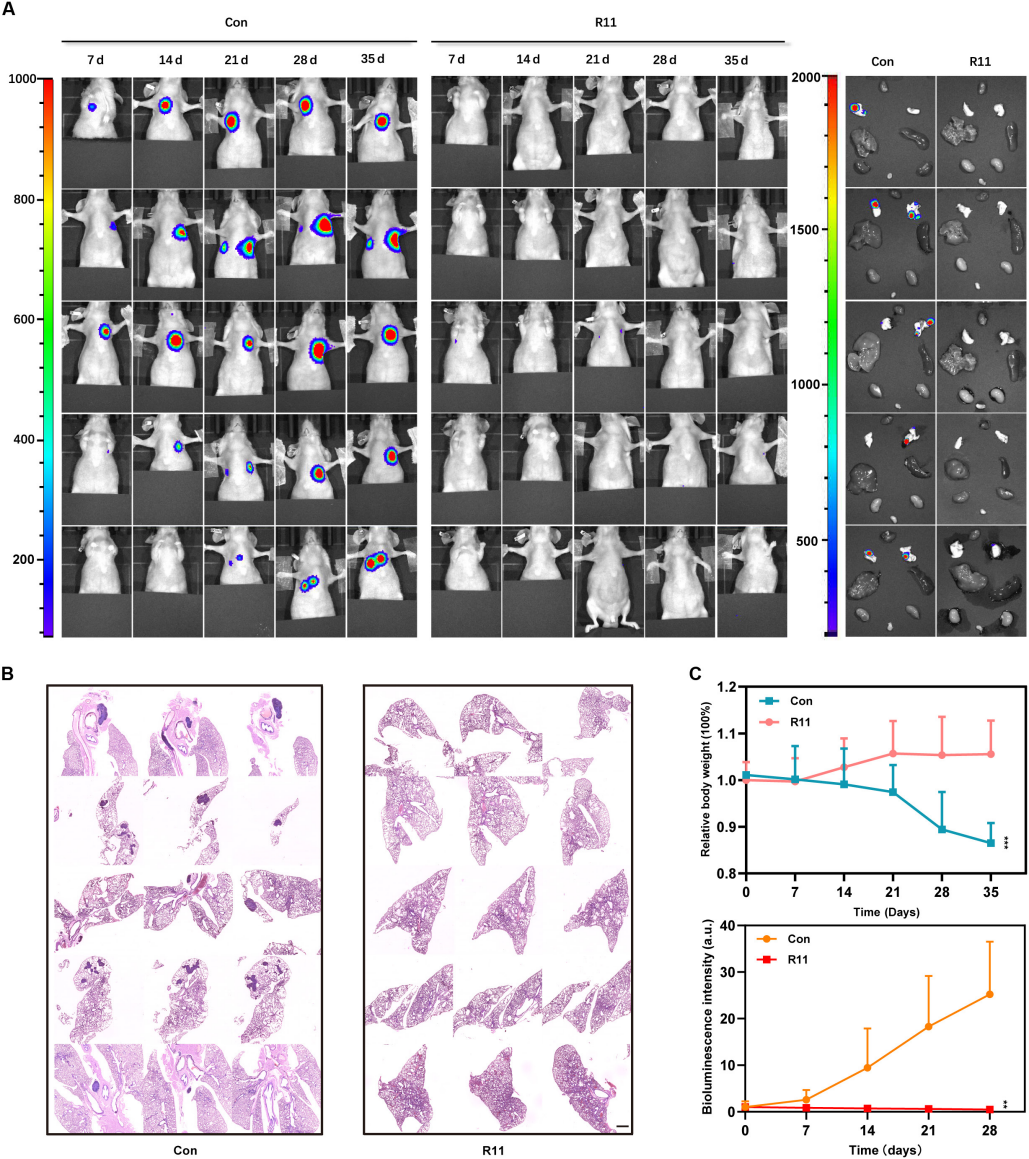
The anti-metastasis effect of R11 in vivo. (A) BCa lung metastasis status in murine models, illustrated by BLI with luciferase–luciferin pairs; T24 cells previously receiving the PBS and R11 (5 μM) were intravenously injected and their lung metastasis niches were monitored every 7 d, with a total of 5 times. The metastasis niches in heart, liver, spleen, lungs, kidney, and bladders, directly imaged by BLI with luciferase–luciferin pairs in vitro. (B) Histopathological analysis of the representative lung tissue from the corresponding mice in (A) by H&E staining. The lungs were sliced from the apex to the base, with an interval between 2 adjacent slides being 200 μm. Scale bar, 1 mm. (C) Average body weight of mice in the corresponding groups in (A). In vivo bioluminescence intensity curves of BCa lung metastasis in the corresponding groups in (A). **P < 0.01; ***P < 0.001.

T24 cells previously treated with R11 at a single dose (0 μM/5 μM) were intravenously injected, and the possibility of lung metastasis was evaluated for a long duration. On the seventh day, the lung metastasis of T24 cells merged in 5 mice from the control group, and the tumor volume increased during the follow-up period. In contrast, a single dose of R11 greatly impaired the lung metastasis ability of T24 cells in vivo, as no luciferin bioluminescence was detected during the whole follow-up period. On the 35th day, the heart, liver, spleen, lung, kidney, and bladder were extracted and sent for histopathological analysis. The sections were observed under a microscope between 2 adjacent slides at 200-μm intervals throughout the whole tissues (Fig. 3A). Consistent with the outcomes of the bioluminescence imaging (BLI), multiple tumors were found in the lungs of mice free of R11 treatment, whereas R11-treated tumor cells did not metastasize to the lungs (Fig. 3B). Weight gain was observed only in the mice in the R11-treated group (Fig. 3C).

### Intracellular target effects of R11 on the cytoskeleton

**Fig. 4.**
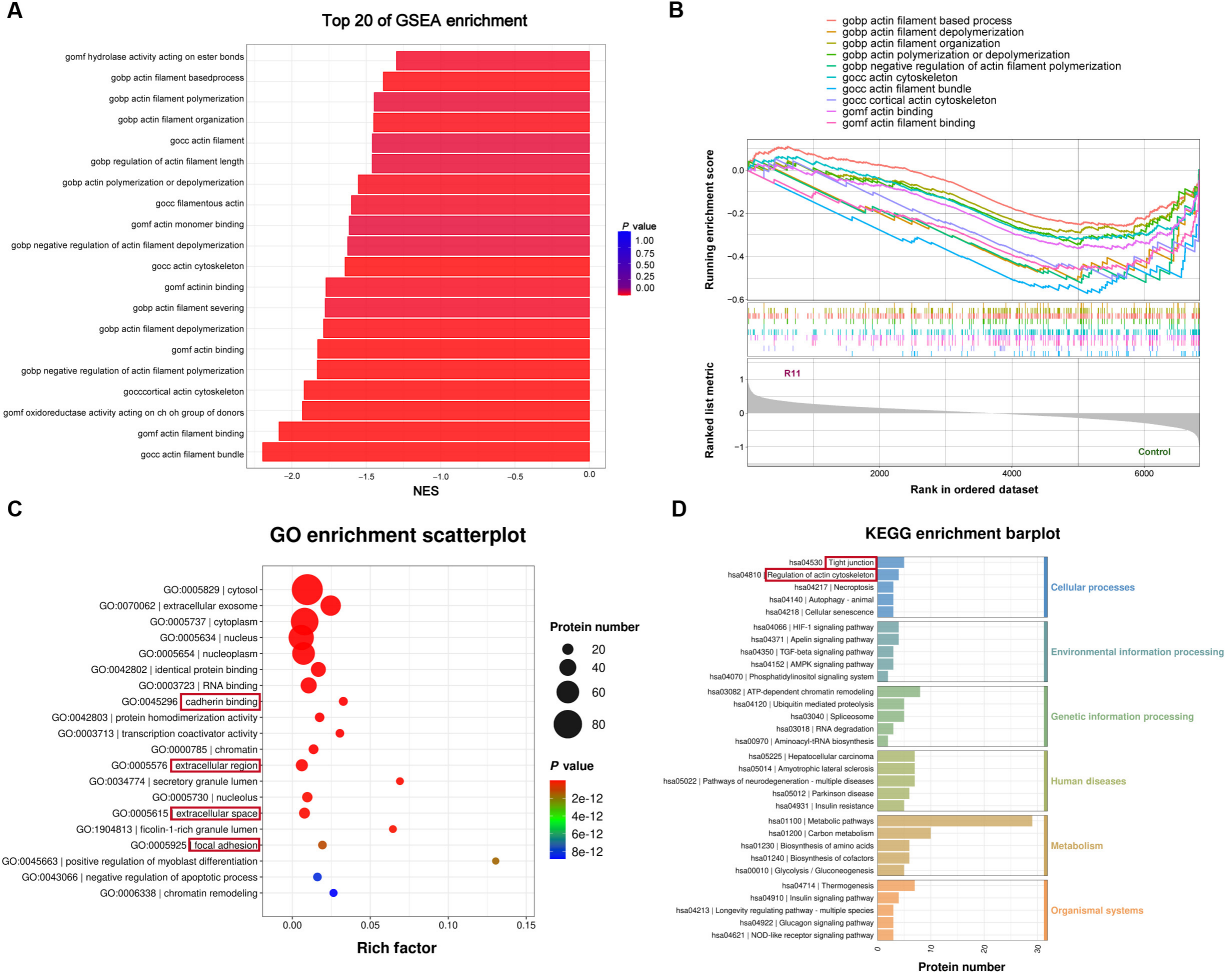
Proteomics analysis of the R11 threat on BCa cells. (A and B) Gene Set Enrichment Analysis (GSEA) enrichment analysis. NES, normalized enrichment score. (C and D) GO and KEGG enrichment analysis.

**Fig. 5.**
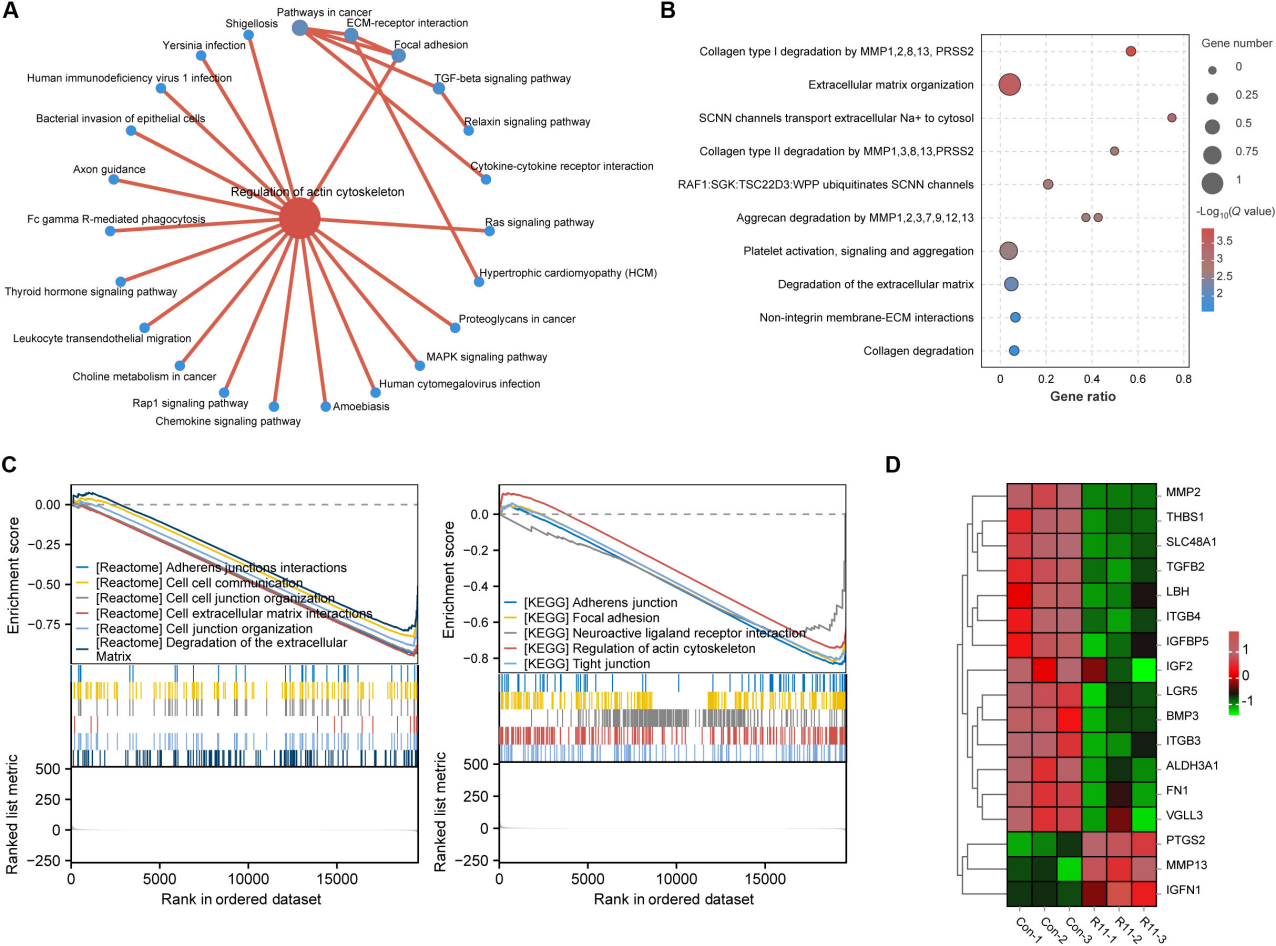
RNA sequencing analysis of the R11 threat on BCa cells. (A) Pathway interaction networks reveal the downstream signaling pathways and physiological processes influenced by the actin cytoskeleton-regulating gene sets. (B) GO analysis. (C) GSEA analysis from the Reactome and KEGG datasets. (D) Heatmap showed expression alterations of ECM-related specific genes after the exposure to the R11 threat.

A key step in BCa metastasis is cytoskeleton remodeling, which subsequently activates cytoskeleton stretching and contraction to achieve cell mobility [20]. Cytochalasin D, an actin polymerization inhibitor, prevents R11 (5 μM) from crossing the BCa cell membrane (Fig. S3), suggesting that the cellular uptake of cell-penetrating peptides (CPPs) is generally considered to be regulated by actin cytoskeleton remodeling [21]. Tandem mass tag (TMT)-based quantitative proteomic analysis revealed important enrichment of proteins associated with actin filament assembly and polymerization after R11 treatment (Fig. 4A). Additionally, the overall signaling pathway analysis demonstrated a marked increase in the activity of actin filament-based processes and actin filament depolymerization after R11 treatment (*P* < 0.05) (Fig. 4B). Overall, R11 plays a vital role in modulating cytoskeletal dynamics. Further analysis of biological function revealed that the most pronounced effect caused by R11 was a change in cytoplasmic protein composition, which is specifically related to cell migration and invasion, including cadherins, components of focal adhesions, and ECM proteins (Fig. 4C and D). A downstream change following unbalanced cytoskeleton recycling, analyzed by transcriptome sequencing (Fig. 5A), revealed the up-regulated expression of 157 genes and down-regulated expression of 146 genes in R11-treated cells (Fig. S1). Our analysis revealed that alterations in adhesion junctions, the ECM, and tight junctions were the most pronounced (Fig. 5B and C), and the mRNA expression of matrix metalloproteinases and integrin family proteins was most strongly inhibited by R11 (Fig. 5D). These findings provide more evidence that R11 inhibits cell migration by disrupting the dynamic balance between actin polymerization and depolymerization, as all these genes, which undergo profound transcriptomic and proteomic changes, serve as key components that correlate cell migration with actin remodeling [22,23]. Additional pathways through which R11 interferes with BCa cell migration are shown in Fig. S2.

### R11 unbalances actin dynamics

**Fig. 6.**
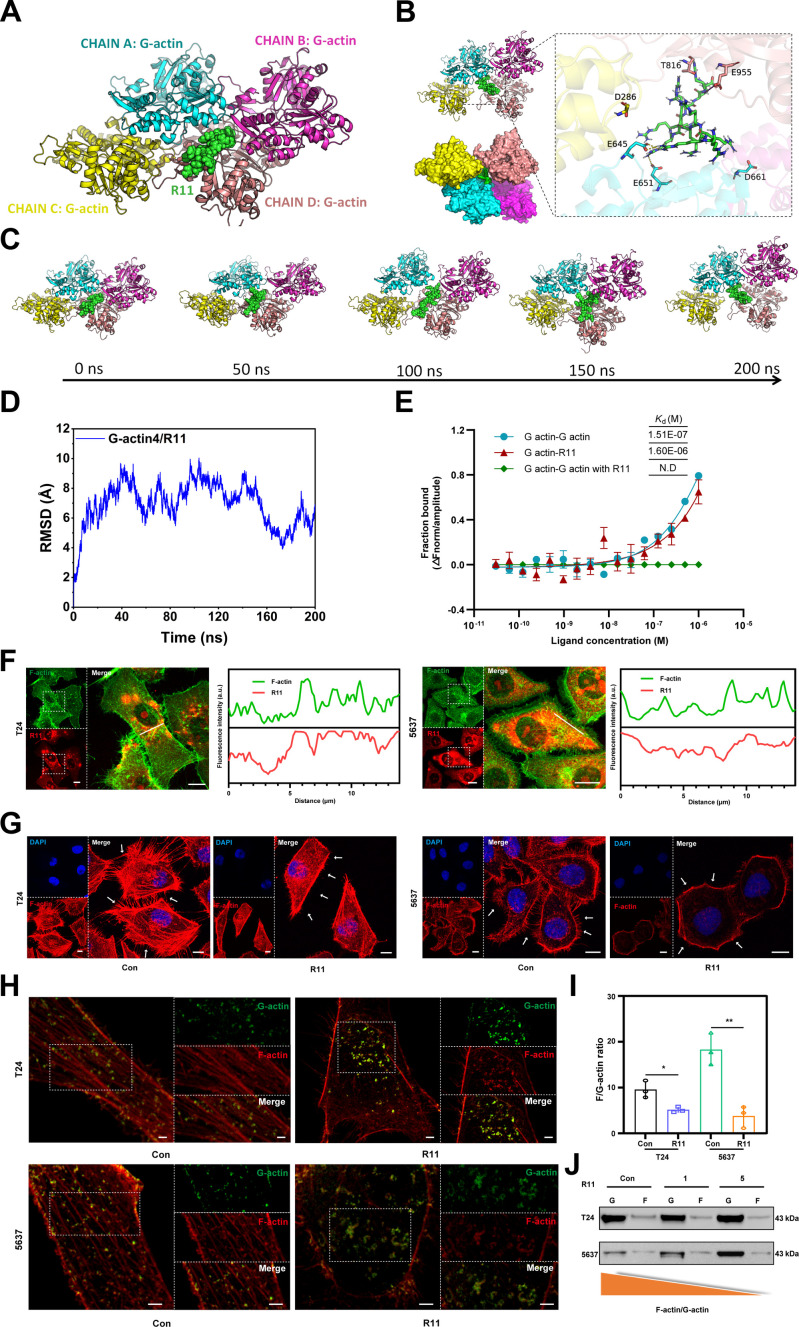
The disruption of actin dynamics by R11. (A) MD stimulation analysis of the actin–R11 interplay. The full-length G-actin monomer crystal structure was obtained from the AlphaFold database, the crystal structure of plectin was downloaded from the PDB database, R11 was constructed based on PyMOL 2.5.5, and the simulation was conducted using AMBER 18 software. (B) R11–actin binding mode at the late phase of their interaction. Yellow dashed lines represent hydrogen bonding, and magenta dashed lines represent salt bridge interaction. (C) Spatial relationship of G-actin tetramer and R11 during the actin–R11 interplay (200 ns), illustrated by the MD analysis. (D) Changes in root mean square deviation (RMSD) during the actin–R11 interplay. (E) Binding affinity of G-actin/G-actin, G-actin/R11, and G-actin (with R11)/G-actin, illustrated by the MST analysis; the *K*_d_ value is calculated by curve fitting; R11 (5 μM). Error bars represented ± SEM (*n* = 3 independent experiments). (F) Intracellular colocalization of R11 and F-actin (left), illustrated by the CLSM analysis; R11 was observed via TAMRA fluorescence channel; F-actin was stained with the fluorescein isothiocyanate (FITC)–phalloidin and observed via FITC fluorescence channel; the TAMRA fluorescence intensity and the FITC fluorescence intensity along the white line in the right inset (right); scale bar, 10 μm; R11 (5 μM), 24-h incubation. (G) Ultra-high-resolution confocal microscopy imaging of F-actin in T24 and 5637 cells; nucleus was observed via DAPI fluorescence channel; F-actin was stained with the TRITC–phalloidin and observed via TRITC fluorescence channel; R11 (5 μM), 24-h incubation; scale bar: 10 μm. (H) Ultra-high-resolution CLSM imaging of G-actin and F-actin. G-actin was labeled with FITC-DBP and observed via FITC fluorescence channel; F-actin was stained with the TRITC–phalloidin and observed via TRITC fluorescence channel; R11 (5 μM), 24-h incubation; scale bar: 2 μm. (I) F-actin/G-actin ratio analyzed by ultra-high-resolution CLSM; *n* = 3 independent experiments. (J) F-actin/G-actin ratio analyzed by the differential sedimentation assay. *P < 0.05; **P < 0.01.

The binding between the guanidino group in the side chain of arginine (Arg^+^) and carboxylates through H-bond-reinforced ionic contacts was shown to interrupt myoglobin aggregation [24]. Studies have also shown that positively charged substances can interact with actin through nonspecific electrostatic adsorption, leading to disturbances in actin dynamics [7]. Many studies have reported the use of cationic polymers for the spatiotemporal interruption of G-actin polymerization [7]. Inspired by this phenomenon, we deduced that the interaction between highly protonated R11 and negatively charged actin possibly followed the same pattern (Fig. 1). Visualized molecular dynamics (MD) simulations were used to elucidate the mechanism governing the interactions between R11 and actin. The formation of hydrogen bonds and salt bridges between R11 and the negatively charged glutamic and aspartic acids of actin allows R11 to insert into the central interstices of the G-actin tetramer, hence disrupting the stability of the tetramer (Fig. 6A and B). Specifically, at the initial phase of the interaction (0 ns), G-actin monomers are in close proximity to their assemblies as tetramers, whose surface R11 lands on. At 50 ns, the tetramers began to collapse, enabling R11 to migrate more closely to the tetramer center. At 100 ns, the tetramers fell apart more severely so that part of R11 could be inserted into the protein–protein interaction interface. At 150 ns, the crevices of the tetramer were occupied by R11, indicating deeper integration (Fig. 6C). Microscale thermophoresis (MST) offers more direct evidence of the interaction between actin and R11. G-actin monomers displayed strong binding affinity, with the *K*_d_ reaching 1.51 nM, whereas R11 presented a stronger binding affinity (*K*_d_ = 1.60 μM) with G-actin monomers. After the integration of R11 into G-actin monomers, the subsequent interaction between G-actin monomers themselves almost disappears, demonstrating that R11 blocks the polymerization of G-actin monomers (Fig. 6E). Intracellular trafficking analysis revealed that R11 shares a close spatial relationship with F-actin in T24 and 5637 cells, although the distribution of R11 expanded to other intracellular regions (Fig. 6F). In the control group, the bundle appearance of F-actin filaments could be observed, with these structures interconnecting and extending to form a network [25]. However, R11 disorganized the cross-linked F-actin network, leaving only short and dim filaments (Fig. 6G). More detailed information on the colocalization of G-actin and F-actin is provided in Fig. 6H. Under no R11 threat, G-actins were able to adhere to bundled F-actin. Once the cells were exposed to R11, the dynamic balance between F-actin and G-actin was disrupted, resulting in the detachment of G-actin (Fig. 6H). As a result, the ratio of F-actin to G-actin was approximately 2 to 3 times lower than that of the control group (Fig. 6I) and decreased more dramatically as the concentration of R11 increased to 5 μM (Fig. 6J). Taken together, the actin rearchitecture induced by the R11 threat is initiated by strong binding between R11 and G-actin, followed by the unsuccessful transition of F-actin into G-actin, which eventually results from the loss of bundled F-actin.

### R11 regulates the actin–plectin–vimentin/ITGβ4 pathway

**Fig. 7.**
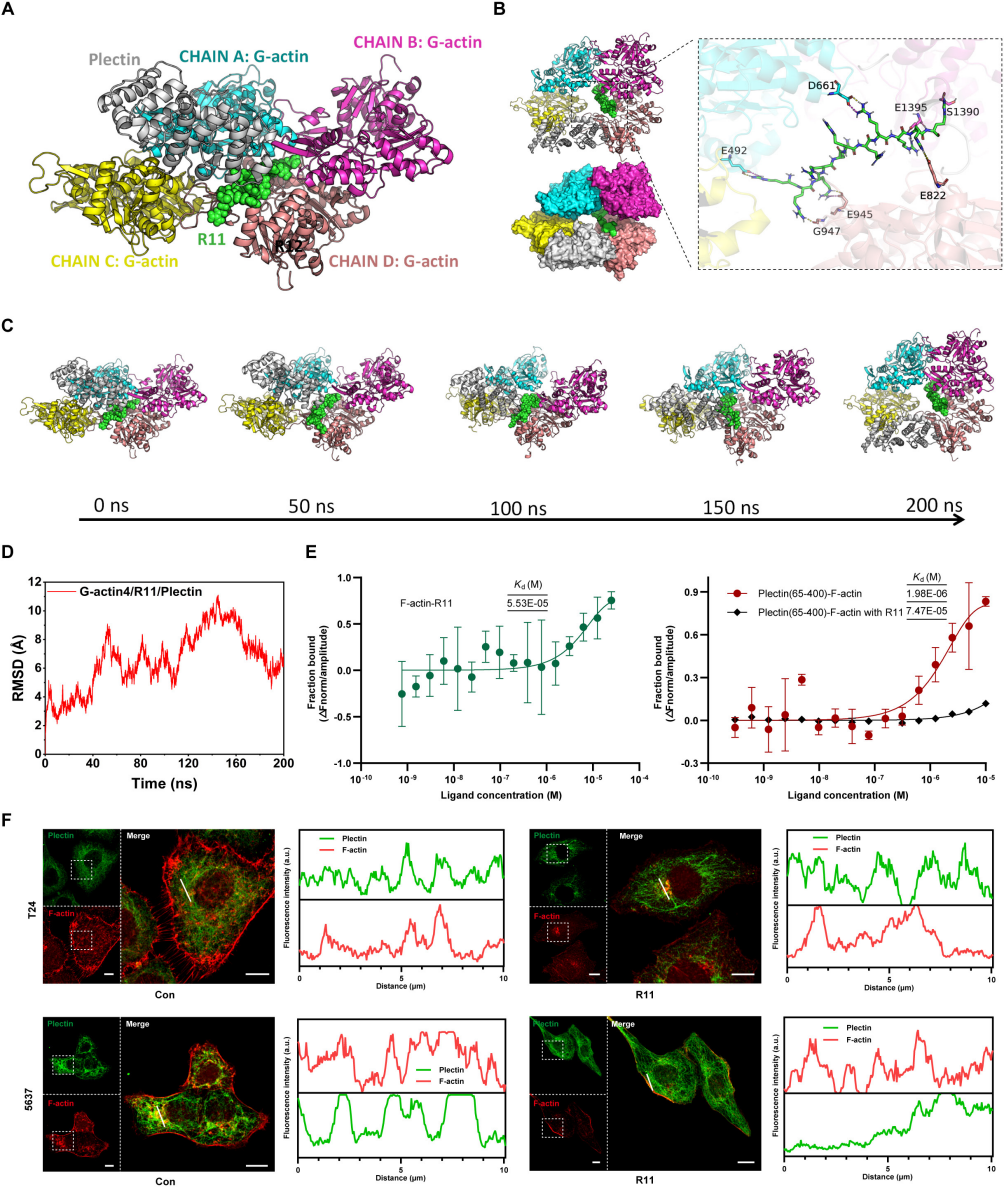
The disruption of the interaction between actin and plectin by R11. (A) MD stimulation analysis of the R11-participated G-actin–plectin interplay. (B) R11-participated G-actin–plectin binding mode at the late phase of their interaction. Yellow dashed lines represent hydrogen bonding, and magenta dashed lines represent salt bridge interaction. (C) Spatial relationship of G-actin tetramer and plectin during the R11-participated actin–plectin interplay (200 ns), illustrated by the MD analysis. (D) Changes in RMSD during the R11-participated actin–plectin interplay. (E) Binding affinity of F-actin/R11, F-actin (with R11)/plectin, and F-actin (with R11)/plectin, illustrated by the MST analysis; the *K*_d_ value is calculated by curve fitting; R11 (5 μM). Error bars represented ± SEM (*n* = 3 independent experiments). More details on the 65-400 binding domain of plectin with actin can be found in UniProt Tools and related literature [39]. (F) Intracellular colocalization of F-actin and plectin (left), illustrated by the CLSM analysis; plectin was observed via Alexa 488 fluorescence channel; F-actin was stained with the TRITC–phalloidin and observed via TRITC fluorescence channel; scale bar, 10 μm; the TRITC fluorescence intensity and the Alexa 488 fluorescence intensity along the white line in the right inset (right); R11 (5 μM), 24-h incubation.

Plectin cross-links 2 filamentous networks, stabilizes the cell–matrix and cell–cell contacts to maintain the integrity of the cytoskeleton, and reorganizes the cytoskeleton during various biological processes, especially cell migration and invasion [26,27]. We hypothesized that the subsequent action triggered by R11–actin interplay might occur between the cytoskeletal linker protein plectin and actin. MD analysis revealed that R11 could be inserted into the G-actin tetramer center and therefore cause dissociation of the G-actin tetramer, hindering plectin binding to the G-actin tetramer. From the beginning of 0 to 50 ns, plectin interacts with chain A and chain C from G-actin, mostly creating a wide area of surface contact between plectin and chain A. When the interaction time reached 100 ns, the interaction hotspot between plectin and G-actin shifted to the area of contact between chain C and plectin. During the late phase of the actin–plectin interplay (200 ns), plectin can only partially bind to chain D of G-actin, causing an unstable connection. Therefore, R11 undermined the stability of plectin binding with the tetramer. In this step, in addition to linking with Glu and Asp residues, R11 also forms hydrogen bonds with serine and glycine residues (Fig. 7A and D). The MST analysis proved that despite the R11–G-actin interaction, R11 also exhibited binding affinity to F-actin, although not as strong as the natural binding affinity (*K*_d_ = 1.98 μM) between plectin (65-400) and F-actin. The introduction of R11 into F-actin nearly completely diminished F-actin/plectin binding (65-400) (Fig. 7E). The intracellular spatial correlation of F-actin and plectin was strong for cells free of R11, but once R11 was applied, F-actin and plectin rarely colocalized (Fig. 7F).

**Fig. 8.**
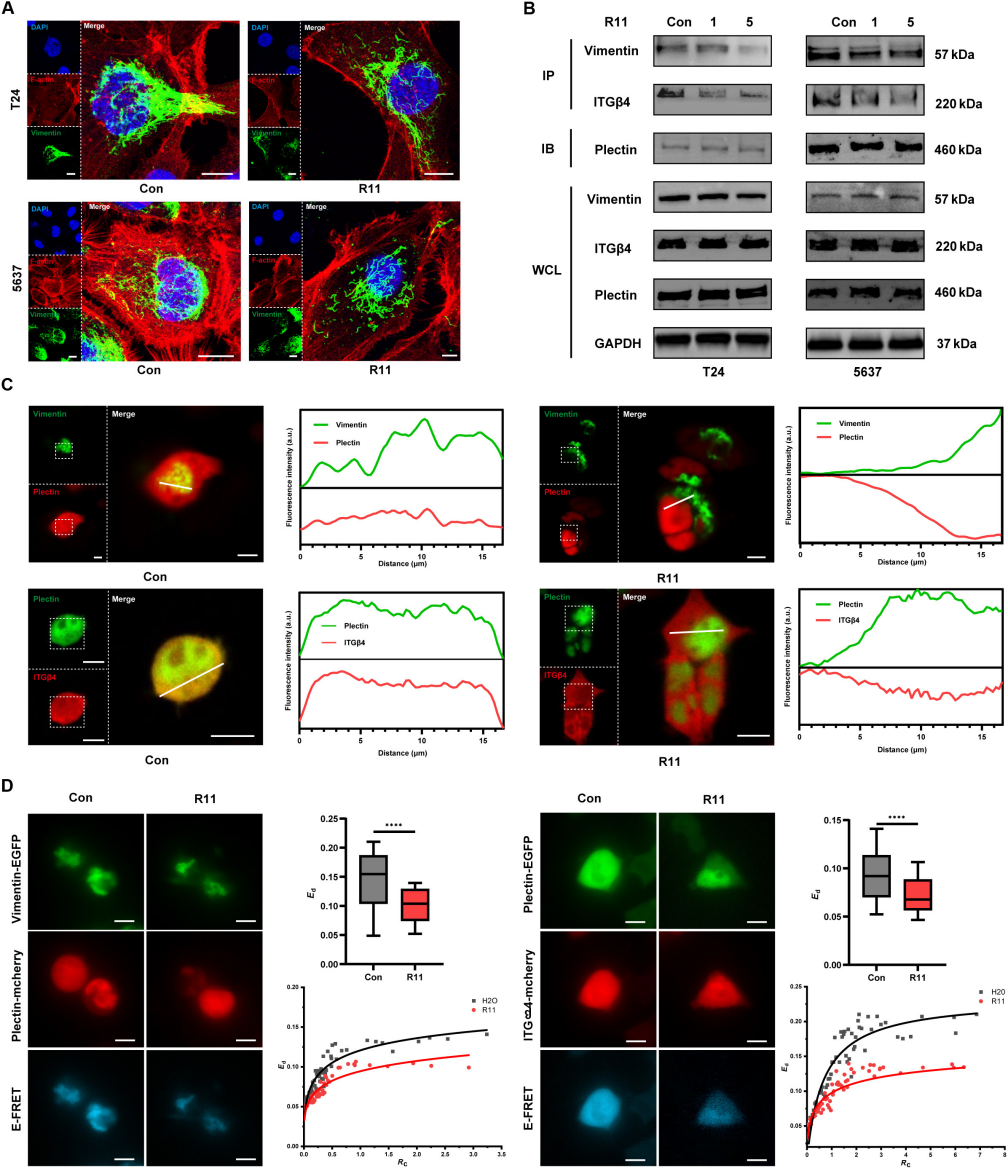
The cascade reaction down the R11–actin–plectin interplay. (A) Intracellular distribution of F-actin and vimentin in T24 and 5637 cells, illustrated by the ultra-high-resolution CLSM analysis; nucleus was observed via DAPI fluorescence channel; F-actin was stained with the TRITC–phalloidin and observed via TRITC fluorescence channel; vimentin was stained with the Alexa 488 fluorescence and observed via Alexa-488 fluorescence channel; R11 (5 μM), 24-h incubation; scale bar: 10 μm. (B) Interactions between plectin and vimentin/ITGβ4, revealed by the immunoprecipitation assay. (C) Intracellular colocalization of vimentin-EGFP and plectin-mCherry (left), plectin-EGFP, and ITGβ4-mCherry (right), revealed by the fluorescence intensity change along the white line drawn in the merged view. Scale bar: 10 μm. (D) Intracellular colocalization and E-FRET of vimentin-EGFP and plectin-mCherry (left), plectin-EGFP, and ITGβ4-mCherry (right), measured by the quantitative FRET analysis in live cells. *E*_d_ values and *E*_d_–*R*_c_ plots representing the efficiency of FRET. Scale bar: 10 μm.

Further insight into the cascade reaction down the actin–plectin interplay was offered. After the addition of R11, the originally polarized vimentin structures around the nucleus became disordered and even dispersed, with the elongated filaments breaking apart (Fig. 8A and Fig. S4). Vimentin and ITGβ4 were much less strongly pulled down by plectin in R11-treated cells, as demonstrated by the coimmunoprecipitation (co-IP) assay (Fig. 8B). F-actin binding to plectin was unchecked because of its poor stability in the co-IP assay. Plectin contains an N-terminal canonical actin-binding domain (ABD) linked to a C-terminal intermediate filament-binding domain (IFBD) through a central rod domain [5], and the ABD serves as a competitive binding site for both the actin and ITGB4 subunits [26,28]. However, owing to the large size of plectin, which complicates its acquisition, MST analysis cannot accurately predict the binding of plectin to vimentin/ITGβ4 following the interaction of R11 with actin. Instead, confocal laser scanning microscopy (CLSM) analysis of the subcellular distribution of different protein pairs in cells was used. Confocal laser scanning microscope (CMSL) colocalization analysis revealed weak spatial correlations between plectin and vimentin/ITGβ4 after R11 infection. Furthermore, live-cell fluorescence resonance energy transfer (FRET) analysis provided more precise insight into the loss of molecular binding between plectin and vimentin/ITGβ4 following R11–actin–plectin interplay (Fig. 8C and D).

### Multivalent R11 assemblies display enhanced actin-disrupting and antimetastatic abilities

**Fig. 9.**
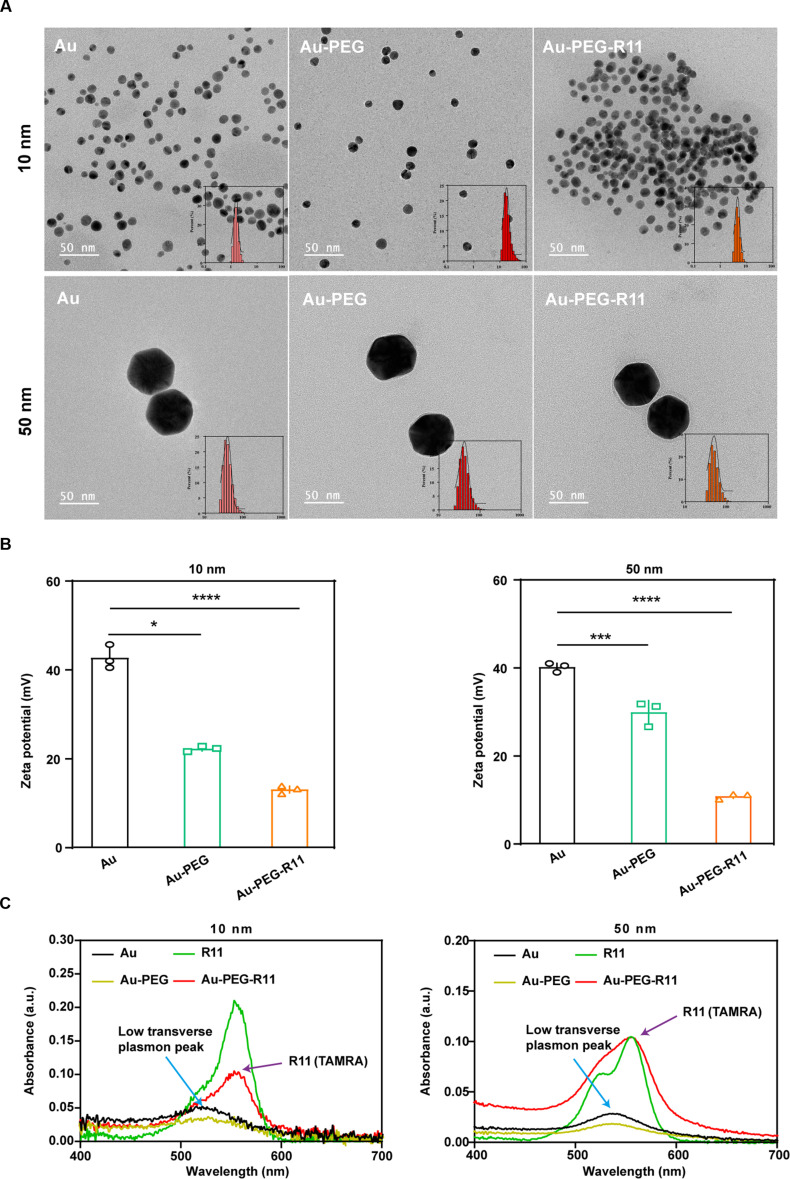
Physicochemical characterization of Au–PEG–R11 NPs. (A) Morphology and size distribution of NPs reveled by the TEM. (B) Zeta potentials of NPs; *n* = 3 independent experiments. (C) UV–Vis absorption spectra of NPs. *P < 0.05; ***P < 0.001; ****P < 0.0001.

Multivalent nanomaterials have emerged as promising platforms in BCa treatment by improving targeted delivery, enhancing therapeutic efficacy, and enabling combination strategies that go beyond conventional chemotherapy and immunotherapy [29,30]. Nanoparticle-based systems can increase drug retention and specificity in bladder tumors, reduce side effects, and facilitate sustained release of therapeutic agents, addressing key limitations of current treatments [31,32]. Notably, recent nanomedicine approaches such as the multifunctional Fe-EGCG@RSL3 platform have demonstrated synergistic induction of ferroptosis combined with tumor microenvironment remodeling, leading to enhanced antitumor activity and improved immunotherapy responses in BCa models [33–35]. These advances illustrate how engineered nanostructures can integrate multiple therapeutic modalities—such as selective tumor targeting, programmed cell death induction, and immune modulation—to more effectively control primary tumors and potentially metastasis, setting the stage for multivalent R11 assemblies to further extend these capabilities. Next, the multivalent effect [36] was exploited to increase the F-actin-disrupting ability of R11. R11 assemblies were constructed by covalently immobilizing a polyethylene glycol (PEG-2000) spacer and then R11 ligands onto gold nanoparticles (AuNPs) of different sizes (Fig. 9A). The naked AuNPs had a uniform and spheroid morphology, with sizes ranging from 6 to 8/50 to 80 nm. The stepwise surface functionalization of AuNPs misted their borders. The zeta potentials of the AuNPs (*d*: 10 nm/50 nm), Au–PEG NPs (*d*: 10 nm/50 nm), and Au–PEG–R11 NPs (*d*: 10 nm/50 nm) were approximately 42/40, 22/31, and 13/15 mV, respectively. A redshift in the ultraviolet–visible (UV–Vis) absorption spectra was observed for the Au–PEG NPs and the Au–PEG–R11 NPs compared with the AuNPs and the Au–PEG NPs, respectively (Fig. 9B and C). The surface coverage density of the R11 ligands was calculated to be 7.00/nm^2^ for the Au–PEG–R11 NPs (*d*: 10 nm) and 13.18/nm^2^ for the Au–PEG–R11 NPs (d: 50 nm) [37]. The denser surface coverage of R11 on the Au_50_–PEG–R11 NPs than that on the Au_10_–PEG–R11 NPs is likely attributed to the less space occupied by each brush-like, rather than mushroom-like, PEG chain implanted on a relatively flatter surface [38].

**Fig. 10.**
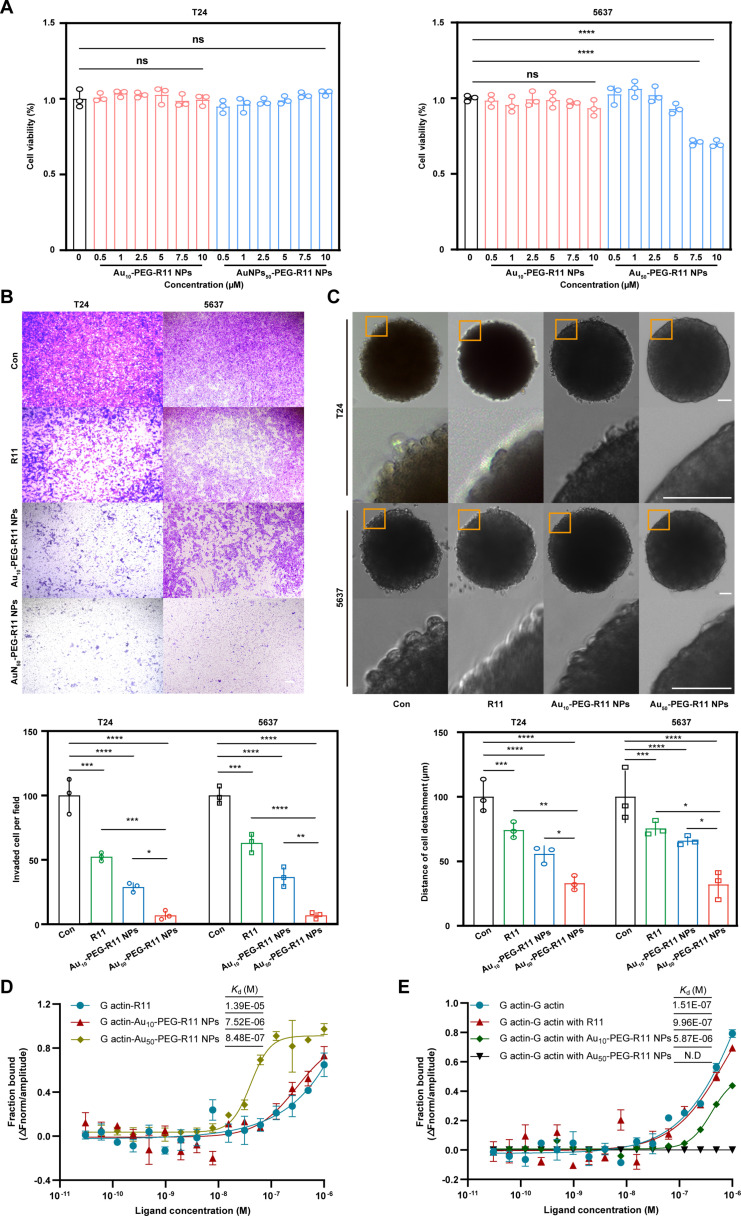
The multivalent R11 assemblies display enhanced actin-disrupting and anti-metastasis ability. (A) Cytotoxicity of cells incubated for 24 h with Au–PEG–R11 NPs at different R11-equivalent concentrations. (B) Effect of different formulations of R11 (equivalent concentration: 1 μM) on the invasiveness of T24 and 5637 cells, determined by Transwell assays; R11 (1 μM), 24-h incubation; *n* = 3 independent experiments; scale bar: 200 μm. (C) Effect of different formulations of R11 (equivalent concentration: 1 μM) on the invasiveness of T24 and 5637 cells, determined by the 3D in-gel spheroid detachment assay; R11 (1 μM), 24-h incubation; *n* = 3 independent experiments; scale bar: 50 μm. (D) Binding affinity of G-actin with different formulations of R11, illustrated by the MST analysis; the *K*_d_ value is calculated by curve fitting; R11 (1 μM). Error bars represented ± SEM (*n* = 3 independent experiments). (E) MST detection of interference with G-actin polymerization by R11, Au_10_–PEG–R11 NPs, and Au_50_–PEG–R11 NPs. The *K*_d_ value represents the molecular binding affinity. The *K*_d_ value is automatically determined by curve fitting; R11 (1 μM). Error bars represented ± SEM (*n* = 3 independent experiments). ***P < 0.001; ****P < 0.0001.

The cytotoxicity profiles of R11 in different formulations were evaluated by the Cell Counting Kit-8 (CCK-8) assay. R11 in different formulations at an equivalent dose of 1 μM caused no toxicity to either BCa cell line unless the concentration exceeded 7.5 μM in the 5637 cell line (Fig. 10A). The cellular uptake of R11 in different formulations was compared under the equivalent R11 (1 μm) supply. The amount of endocytosed R11 anchored onto the Au_50_–PEG–R11 NPs was less than or equal to that anchored onto the Au_10_–PEG–R11 NPs (Fig. S5). As depicted in Fig. 10B and C, only approximately 10% of the cells crossed the membrane insets in the Transwell chambers after receiving a 24-h treatment with the Au_50_–PEG–R11 NPs, approximately 90%, 35%, and 15% less than those in the control group, the R11 group, and the Au_10_–PEG–R11 NP group, respectively. Furthermore, a clear and smooth border was present only in the Au_50_–PEG–R11 NP-treated MCSs, resulting in outer detached layers that were 75%, 50%, and 30% less thick than those in the control group, the R11 group, and the Au_10_–PEG–R11 NP group, respectively (Fig. 10B and C). The more densely R11 assembled, the stronger the anti-metastasis effect it produced. Previously, our study revealed the key role of R11–actin interplay in the antimetastatic mechanism. In this section, MST analysis was used to provide direct evidence that the Au_50_–PEG–R11 NPs eliminated the intermolecular interaction of G-actins, and the actin-disrupting effect contributed by the Au_10_–PEG–R11 NPs was much weaker but approximately 2-fold greater than that induced by free R11 (Fig. 10D and E). Therefore, we believe that multivalent R11 assemblies have a greater influence on actin depolymerization than R11 monomers do.

Taken together, we believe that multivalent R11 assemblies offer distinct advantages in terms of targeting specificity, multivalency, and stability. R11 has been shown to specifically target BCa, and its effect on actin is fundamentally different and more tunable compared to other actin-disrupting agents. R11 demonstrates significantly enhanced actin-disrupting potency through avidity-driven cooperative binding with G-actin. This nanoscale multivalency induces a level of actin destabilization that exceeds the capabilities of monomeric peptides or conventional small molecules while also providing improved structural stability and potentially prolonged intracellular retention, thus enhancing the durability of its cytoskeletal modulation.

## Conclusion

The effectiveness of current actin-targeting therapies is reduced by their lack of clear functional sites and off-target effects. In this work, the bladder tumor-targeting and cell-penetrating polyarginine peptide R11 was employed to disrupt actin dynamics and induce the domino effect on the actin–plectin–vimentin/ITGβ4 pathway. As a result, the whole cytoskeleton was disorganized, and tumor metastasis was inhibited. Such actin-disrupting and antimetastatic effects could be further enhanced once R11 was presented as a multivalent assembly.

To improve R11’s clinical potential, future strategies should focus on optimizing its delivery. Nanoparticle formulations, such as PEGylated or gold nanoparticles, could enhance its tumor targeting and minimize systemic toxicity. Local delivery methods, such as intravesical instillation or inhalable aerosols, may provide better tumor site concentration and reduce off-target effects. Furthermore, combining R11 with other therapies, such as chemotherapy or immune checkpoint inhibitors, could strengthen its anti-metastatic effects and address resistance to single-agent treatments. Careful consideration of potential immune responses will also be necessary to ensure the peptide’s long-term effectiveness and safety. In summary, R11 shows strong potential as a targeted therapeutic agent for BCa and other cytoskeleton-dependent cancers, with further optimization of delivery systems and combination therapies required for clinical translation.

## Materials and Methods

### Materials

R11 (GRRRRRRRRRRR) and Rhodamine-R11 were purchased from GL Biochem. Ltd. (Shanghai, China). Hexadecyl trimethyl ammonium bromide-modified gold nanoparticles (CTAB-Aunp) were purchased from XFNANO Ltd. (Jiangsu, China). HS-PEG2000-NH_2_ was purchased from Tansh-Tech Ltd. (Guangzhou, China). Tris (2-carboxyethyl) phosphine (TCEP), 1-(3-dimethylaminopropyl)-3-ethylcarbodiimide (EDC), and N-hydroxy succinimide (NHS) were purchased from Sigma (St. Louis, MO, USA). 2-Morpholinoethanesulfonic acid (MES buffer pH 6.7) was obtained from Macklin Biochemical Co. Ltd. (Shanghai, China). The following compounds were procured from Thermo Fisher Scientific (Waltham, MA, USA): fetal bovine serum (FBS), penicillin–streptomycin solution, RPMI 1640 medium, Dulbecco’s modified Eagle’s medium (DMEM), and trypsin–EDTA. We bought CCK-8 from Yeasen Biotech Co. Ltd. (Shanghai, China).

### Cell culture

The 5637 cells were grown in RPMI 1640 media at 37 °C with 5% CO_2_, while the T24 cells were cultivated in DMEM with 10% (v/v) FBS and 1% (v/v) penicillin–streptomycin. The Chinese National Collection of Authenticated Cell Cultures was the source for the cell lines used in this study.

### Cellular distribution of R11

In laser confocal Petri plates, 10^5^ cells per microplate were used to seed T24 and 5637 cells, respectively, and then let to incubate for 24 h. The cells were exposed to 5 distinct doses of Rho-R11 for 24 h. Afterward, the samples were moved to a CLSM for examination. The fluorescence was quantified using the ImageJ program.

### In vitro cytotoxicity assay

Preincubation was done for 12 h after seeding T24 and 5637 cells into 96-well plates at a density of 5,000 cells per well. To determine dose-dependent cytotoxicity, the R11 peptide was introduced at 4 different gradient concentrations and left to incubate for another 24 h. Then, in each well, a 10% CCK-8 solution was allowed to sit for 2 h. After that, measurement of the 450-nm absorbance was carried out on a microplate spectrophotometer. Each data point reflects the average of 4 separate biological studies and was subjected to a 5-part analysis.

### Wound-healing assay

T24 and 5637 cells were seeded in 6-well plates (8 × 10^5^ cells per well) and incubated until 100% confluence was reached. Confluent cells were scraped with a 200-μl pipette tip to create a scratch wound. Nonadherent cells were washed away, the remaining cells were treated with R11, and wound healing was evaluated at 0, 12, and 24 h. The migration distance of the cells was calculated by subtracting the initial wound width from the width at 24 h.

### Transwell assay

The top chambers of Transwell plates with 8.0-μm hole polyester membrane inserts (Corning, Thermo Fisher Scientific Inc., Waltham, USA) were used to seed T24 cells and 5637 cells at a density of 5 × 10^5^ cells per well, while the bottom chambers were filled with full cell culture medium. After the culture had been cultured for 24 h, 3 different doses of R11 were added and left to incubate for another 24 h. Before staining the cells on the bottom of the top chamber with 0.1% crystal violet for 10 min at room temperature, the cells on top of the filter were removed and fixed with 4% formalin. A Japanese inverted microscope (TS 100, Nikon Ti) was used for cell counting.

### 3D tumorsphere growth and invasion assay

When making the 3D spheres, T24 and 5637 cells were deployed. Using the drop-hanging technique, 3D spheres were produced. At a density of 1 × 10^6^ cells/ml, the cells were suspended in a mixture of DMEM and RPMI 1640 with 0.12% (w/v) methylcellulose added. After dropping 25 μl of the cell suspension onto the inside of a cell culture plate’s lid, the lid was then turned upside down. A 10-ml solution of PBS buffer (pH 7.4) was poured to the plate to provide a moist environment for the droplets. Moving the dense 3D spheres to a low-adhesion 24-well plate and letting them develop for an additional 72 h was the next step. The EVOS M7000 microscope was used to view the 2 groups after 24 h of treatment with sterile water and 5 μM R11 in an FBS-free medium.

### Inhibitory efficacy of R11 in a murine BCa lung metastasis model

The Zhejiang Provincial People’s Hospital Laboratory Animal Management Committee gave its stamp of approval to all animal treatments (20240218154141325220). All animal procedures were performed according to the guidelines of the Administration Committee of Experimental Animals in Zhejiang Province and the Ethics Committee of Zhejiang Provincial People’s Hospital. Luciferase-labeled T24 cells were used to establish a cancer metastasis model. Briefly, ten 4-week-old nude mice were randomly divided into 2 groups and acclimatized for 7 d. After that, we put the mice on a heated platform and gave them a 1% isoflurane/oxygen inhalation to make them unconscious. In the end, a 28-gauge insulin needle was used to inject 2 × 10^6^ luciferase-labeled T24 cells in 200 μl of culture media into the tail vein. BLI was then carried out weekly for 35 d to track distant metastases. A 150 mg/kg intraperitoneal injection of d-luciferin was given before each imaging session. The mice were put down, and their lungs and any other organs thought to have metastasized were removed at the conclusion of the experiment. Quantification was done on the surface of the lung for metastatic nodules. After that, the removed lungs were stained using hematoxylin and eosin (H&E) and immunohistochemistry.

### TMT-labeled quantitative proteomics

Proteomic analysis was carried out by LC-Bio Technologies of Hangzhou, China, after T24 cells were exposed to 5 μM sterile water and R11 for 24 h. The workflow for quantitative proteomic analysis involved 7 main stages, including protein extraction and validation, digestion, TMT labeling, high-pH reversed-phase high-performance liquid chromatography (HPLC) fractionation, nano-liquid chromatography–tandem mass spectrometry (LC-MS/MS)-based quantification, and final protein identification. To summarize, the proteins were isolated by first homogenizing the tissue using a tissue lyser at 60 Hz for 2 min and then centrifuging at 20,000*g* for 15 min at 4 °C. The Bradford technique and sodium dodecyl sulfate–polyacrylamide gel electrophoresis (SDS-PAGE) were used for quality control of the protein extraction process. After that, a 50:1 ratio of protein to trypsin was used to digest the proteins that were produced. The peptides were tagged with TMTs (Thermo Fisher Scientific, USA) after digestion. A 3.5 μm 4.6× 150 mm Agilent ZORBAX 300Extend-C18 column, attached to an UltiMatem 3000 binary fast separation system (Thermo Fisher Scientific, USA), was used to combine and fractionate peptides in equal amounts from each sample. The fractions were collected every minute throughout the gradient elution, which was carried out by watching the elution peaks at 214 nm. Fractions were freeze-dried after extraction. After that, the EASY-nLCm 1200 system (Thermo Fisher Scientific, USA) was used to resuspend and separate the dried peptide samples. After being ionized with a nanoelectrospray ionization (nano-ESI), the isolated peptides were sent for data-dependent acquisition (DDA) mode detection to an Orbitrap Explorism 480 mass spectrometer (MS; Thermo Fisher Scientific, USA). The raw data from TMT-plexed MS/MS analyses were performed using MaxQuant (version 2.1.4.0). We used R (version 4.0.0) to do the statistical analysis. When both the *P* value and the fold change were less than or equal to 0.05 and 1.5, respectively, proteins were deemed substantially different. In order to further analyze the data, heatmaps, principal components analysis charts, volcano plots, and scatterplots for biological process enrichment were created using the OmicStudio tools, which may be found at https://www.omicstudio.cn/tool.

### RNA sequencing and bioinformatics analysis

Following the instructions provided by the manufacturer, total RNA was extracted using a TRIzol reagent kit from Invitrogen (Carlsbad, CA, USA). Agilent Technologies’ 2100 Bioanalyzer and ribonuclease (RNase)-free agarose gel electrophoresis were used to evaluate the RNA quality. Using oligo(dT) beads, eukaryotic messenger RNA was isolated after total RNA extraction. New England Biolabs (Ipswich, MA, USA) (NEB#7530) provided the NEBNext Ultra RNA Library Prep Kit for Illumina, which was used to reverse-transcribe the enriched mRNA into cDNA after fragmenting it into small pieces using fragmentation buffer. A base was added to the purified double-stranded cDNA fragments, and then they were ligated to Illumina sequencing adapters after being end repaired. Utilizing AMPure XP beads (1.0X), the ligation reaction mixture underwent purification. Not only that, but polymerase chain reaction (PCR) amplification was also carried out. Gene Denovo Biotechnology Co. Ltd. (Guangzhou, China) used the Illumina NovaSeq 6000 platform to sequence the resultant cDNA library. In order to identify the genes that were differentially expressed, we used DESeq2 (1.24.0) analysis to compare the global transcript profiles of the control and R11 groups. Genes that fulfilled the filter conditions—a *P* value of less than or equal to 0.05 and a |log_2_-fold change| greater than or equal to 2—were classified as difference expression genes (DEGs). Then, with a *P* value of <0.05, we ran Reactome analysis, Kyoto Encyclopedia of Genes and Genomes (KEGG) enrichment, and Gene Ontology (GO) word analyses. We used the Omicsmart platform (https://www.omicsmart.com/) to do the bioinformatic analysis.

### Immunofluorescence

Laser confocal Petri plates were used to seed T24 and 5637 cells individually, with 10^4^ cells per microplate. The cells were then treated for 24 h. T24 and 5637 cell lines were examined for subcellular colocalization and morphology of R11, F-actin, plectin, and vimentin after being treated with 5 μM R11 or Rho-R11 for 24 h. Cells were permeabilized with 0.5% Triton X-100, fixed with 4% formalin, and washed 3 times with PBS (pH 7.4). Prior to incubation with the specified primary antibodies at 4 °C overnight, the cells were blocked for 30 min in a solution containing 1% bovine serum albumin (BSA) and 5% normal goat serum (v/v). After a pH 7.4 cell wash the next day, the cells were treated for 1 h with secondary antibodies labeled with Alexa Fluor 488, 594, and 647 (Thermo Fisher Scientific, USA). After washing the cells, 4′,6-diamidino-2-phenylindole (DAPI) (Yeeasen Biotech, Shanghai, China) was used to seal them. For this investigation, we used the following antibodies: D6A11 lectin and D21H3 vimentin XP. Cell Signaling Technology (Danvers, MA, USA) supplied the rabbit monoclonal antibodies (Alexa Fluor 488 conjugate). F-actin was stained with SF488–phalloidin (CA1640, Solarbio) and tetramethyl rhodamine isothiocyanate (TRITC)–phalloidin (CA1610, Solarbio).

### Analysis of actin cytoskeletal conformational shifts

Prior to that, fluorescent tags for the G-actin-specific binding protein Gc-globulin (G8764, Sigma) were applied using the Alexa Fluor 488 Microscale Protein Labeling Kit (A30006, Invitrogen, Thermo Fisher Scientific). With the use of phalloidin, F-actin was marked. The cells were then exposed to R11 (5 μM) for a duration of 24 h. After 3 washes with PBS (pH 7.4), the cells were fixed with 4% formalin. Before being rinsed 3 times with PBS (pH 7.4), fixed cells were treated with 0.5% Triton X-100. After being rinsed 3 times with PBS (pH 7.4), the cells were blocked for 30 min in a solution containing 1% BSA and 5% normal goat serum (v/v). Alexa Fluor 488-DBP (Ge-globulin from human plasma) was added to the samples and left to incubate overnight at 4 °C. Next, a 30-min incubation with TRITC–phalloidin (CA1640, Solarbio) the next day, the nuclei were stained with DAPI (40728ES03, Yeasen Biotech, Shanghai, China). A ​STELLARIS 8 STED microscope​ (Leica Microsystems, Germany) in STED and ​lightning mode with excellent lateral and axial resolution was used to collect samples in order to clearly detect the polymerization of G-actin into F-actin filaments. To obtain high signal-to-noise ratios, images were improved using Lightning AI-based deconvolution (LAS X, v3.7).

### Microscale thermophoresis

The interaction between R11 and G-actin/plectin/vimentin/ITGβ4 was determined using an NT.115 assay. The experiment was conducted at 25 °C using a Monolith instrument from NanoTemper Technologies with standard glass capillaries from the same company (catalog no. MO-K022). Purified from HEK293T cells, the recombinant proteins G-actin/vimentin/plectin/ITGβ4 served as a source for targets that were fluorescently tagged. Following the manufacturer’s instructions, G-actin, plectin, vimentin, and ITGβ4 were tagged with Cy5 using a Monolith RED-NHS 2nd Generation Protein Labeling Kit (NanoTemper Technologies). To eliminate the precipitates, all samples that were going to be analyzed were dialyzed against MST buffer, which is a PBS solution that contains 0.01% Tween 20. After that, they were centrifuged at 12,000*g* for 15 min. All the dissociation constants (*K*_d_ values) were obtained by fitting the binding curve with the *K*_d_ model method in MO Affinity Analysis v2.3 software (NanoTemper Technologies).

### Coimmunoprecipitation

We followed the manufacturer’s method to do a co-IP using a Pierce Classic Magnetic Beads IP/Co-IP Kit (88804, Thermo Fisher Scientific). To summarize, Pierce Protein A/G Magnetic Beads were attached to plectin (D6A11, Cell Signaling Technology) antibody for 30 min. Using antibody beads and an overnight incubation at 4 °C with rotation, the cell lysates were treated with total protein. The immunoprecipitates were then submitted to SDS-PAGE and standard Western blotting methods after the bound antigen was eluted. Cells were lysed using cold IP lysis buffer (Pierce, 87788, Thermo Fisher Scientific). After denaturation, 20 μg of protein per sample was resolved by electrophoresis on 4% to 20% gradient precast SDS-PAGE gels (SurePAGE, M00656, GenScript, Nanjing) prior to transfer to a polyvinylidene difluoride (PVDF) membrane (Merck Millipore, Germany). The membranes were blocked for 2 h with 10% nonfat milk and then treated with the specified antibodies at 4 °C for the night. Afterward, secondary antibodies coupled with horseradish peroxidase (HRP) were used to detect the immunocomplex. The proteins may be better seen using fluorography with the use of chemiluminescence detection (ECL, Bio-Rad, California, USA). Cell Signaling Technology (Danvers, MA, USA) supplied the antibodies utilized in the experiment, which included glyceraldehyde-3-phosphate dehydrogenase (GAPDH) (D16H11), vimentin (D21H3), ITGβ4 (D8P6C), and an HRP-linked antibody [anti-rabbit immunoglobulin G (IgG), 7074].

### Measurement of the cellular G- and F-forms of actin

F-actin and G-actin levels were measured using a G-actin/F-actin In Vivo Assay Kit (BK037, Cytoskeleton) according to the manufacturer’s instructions.

### Molecular dynamics

The full-length G-actin monomer crystal structure was downloaded from the AlphaFold database (https://alphafold.ebi.ac.uk/), and by referring to the crystal structure with the Protein Data Bank (PDB) ID 3lue, a G-actin tetramer was obtained through alignment using PyMOL 2.5.5. The crystal structure of plectin was downloaded from the PDB database with PDB ID 4q59. R11 was constructed with PyMOL 2.5.5. Furthermore, R11 and the G-actin tetramer were placed together to obtain G-actin4/R11, and R11, the G-actin tetramer, and plectin were placed together to obtain G-actin4/R11/plectin, respectively, to obtain the initial simulation conformations to explore the impact of R11 on the aggregation of the G-actin tetramer and the interaction between the G-actin tetramer and plectin. The AMBER 18 program was used to conduct all-atom MD simulations using the composite model indicated before as the starting point. An analysis of the protein was conducted using the ff14SB protein force field prior to simulation. The LEaP module was used to include hydrogen atoms into the system, incorporate an octahedral TIP3P solvent box with a 10-Å cutoff distance into the system, balance the system charge using Na^+^/Cl^−^, and lastly generate the simulation’s topology and parameter files.

### Construction of Au–SH–PEG2000–NH_2_–R11 NPs of different sizes

First, 0.55 mM SH-PEG2000-NH_2_ was added to disulfide bond reduction buffer containing 1.35 mM TCEP. After 1 h of disulfide bond reduction, 0.05 mg/ml aqueous solutions of AuNPs with sizes of 10 and 50 nm were added. The Eppendorf (EP) tube was wrapped with aluminum foil and placed on a rotator for mixing at room temperature overnight. The above solution was centrifuged at 800 rpm, the supernatant was collected, and ultrasonication was used to obtain uniformly dispersed Au–PEG2000–NH_2_ NPs. Next, 2 mM Rho-R11, 0.1 M EDC, and 5 mM NHS MES buffer (pH 6.0) were added to the above mixture, and an appropriate amount of 1% Tween 20 was added to stabilize the system. The EP tube was wrapped with aluminum foil and placed on a rotator for mixing at room temperature for 2 h. Finally, gradient centrifugation was performed 3 times using ultracentrifuge tubes and redispersed in ultrapure water, after which ultrasonication was used to obtain uniformly dispersed 10- and 100-nm Au–PEG2000–R11 NPs. The above-synthesized series of Au–PEG2000–R11 NPs was treated with lysis buffer containing 1.5 M dithiothreitol (DTT) and 0.4 M NaCl, and the R11 concentration on the surface of AuNPs of each size was accurately quantified using a standard curve. The R11 standard curve was quantified by BioTek detection.

### Characterization of nanoparticles

The zeta potential and size distribution were determined using a Zetasizer instrument (Malvern Zetasizer, Malvern Ins. Ltd., Malvern, UK). The morphologies were observed using a transmission electron microscope (TEM; FE, JEM-F200, Japan). The UV–NIR (near-infrared) absorption spectrum was obtained from a UV–Vis spectrophotometer (Thermo Fisher Scientific, EVOLUTION220, USA).

### Quantitative FRET imaging and *E*_d_ saturation assay

Confocal dishes were seeded with 200 μl of T24 cells (1 × 10^4^ cells per well) and left to grow for 24 h. Following the manufacturer’s procedure, Turbofect transfection reagent was used to transiently transfect plasmids into the cells. Using a wide-field fluorescence microscope, quantitative FRET imaging was carried out. The raw pictures of the enhanced green fluorescent protein (EGFP), E-FRET, and mCherry channels were acquired using separate excitation/emission filters. At BP488/10 nm, the donor excitation channel activated the EGFP fluorescence, and at BP525/40 nm, the donor detection channel detected it. The donor excitation channel (BP488/10 nm) was used to trigger the E-FRET fluorescence, while the acceptor detection channel (BP610/10 nm) was used to detect it. A fluorescence signal corresponding to mCherry was induced in the BP561/10 nm acceptor excitation channel and detected in the BP610/10 nm acceptor detection channel. Cotransfection of several doses of mCherry-tagged protein plasmid with 300, 900, and 1,800 ng of EGFP-tagged protein plasmid was performed on the cells. Following the procedures outlined before, fluorescent imaging was carried out. Starting with the following function, the saturation binding curves were fitted: The expression for *E*_d_ is: *E*_dmax_ × *R*_c_/(*K*_d_ + *R*_c_). When the acceptor’s donor binding sites are saturated, the maximum energy density (*E*_dmax_) is reached.

### Inductively coupled plasma–mass spectrometry

The masses of the samples and sample tubes were weighed and recorded. One milliliter of aqua regia was added to the sample tubes in multiple aliquots, and the sample was washed with aqua regia in the digestion tubes. After the sample tube was air-dried, the mass of the sample tube was measured again. The difference in mass between the 2 measurements represents the mass of the sample. The sample digestion containers were placed in a microwave digestion system for sample digestion. The samples were digested using a preset temperature program. The digestion process was carried out at 100 °C for approximately 30 min. After digestion, approximately 0.5 ml of digestion solution remained. The digestion tubes were removed and allowed to cool to room temperature in a fume hood. The digestion solution was transferred and filtered into a 10-ml volumetric flask, and the digestion container was rinsed 3 times with deionized water. The solution was then diluted to the mark, mixed thoroughly, and stored for analysis. The samples were analyzed using a PerkinElmer NexION 300X.

Calculation of R11 loading on gold nanospheres and the cellular uptake of R11: The loading of R11 on gold nanospheres can be calculated using the molar mass of gold, the radius and surface area of the nanospheres, the lattice constant of gold, and the concentration of gold measured by inductively coupled plasma (ICP)–MS in combination with the standardized concentration of R11 (1 μM). Furthermore, the amount of R11 taken up by cells can be determined using the above results and the following equation.

$$\frac{\mathrm{Au}\;\text{content in the standard}}{R11\;\text{content in the standard}}=\frac{\text{Cellular uptake content of}\;\mathrm{Au}}{\text{Cellular uptake content of}\;R11}$$ (1)

### Statistical analysis

The data are expressed as the means ± standard deviations (SDs). Significant differences were determined using Student’s *t* test or one-way analysis of variance (ANOVA), as appropriate. A 2-tailed *P* value of <0.05 was considered statistically significant. *P < 0.05; **P < 0.01; ***P < 0.001; ****P < 0.0001. All the data were analyzed with GraphPad Prism version 10.0 (GraphPad Software, CA, USA).

## Data Availability

All essential data are provided within the manuscript and its supplementary materials, and further information is available from the corresponding authors on request.
