# Supplementary material for: Polyarginine Peptide R11–Actin Interaction Induces a Domino Effect on Cytoskeleton Remodeling to Suppress Bladder Cancer Metastasis
Source: Research (Wash D C). 2026 Jan 29;9:1109. doi: 10.34133/research.1109 (PMC12852570; doi:10.34133/research.1109)
Supplement: Supplementary 1 — Figs. S1 to S5 [file research.1109.f1.zip › Supplementary Material.docx]

**Supplementary Information
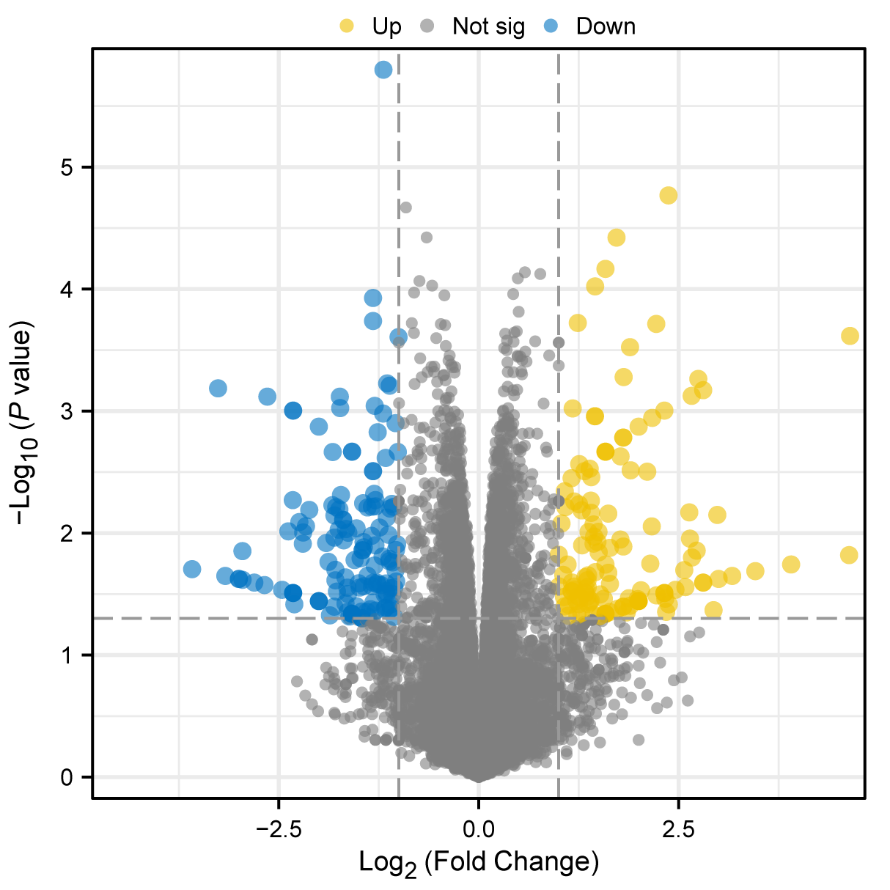
**

**Fig. 1.** Volcano plot illustrating differentially expressed genes (DEGs) in R11 treated group and control group, with threshold as |logFC| > 1 & *p*.adj <0.05.

**
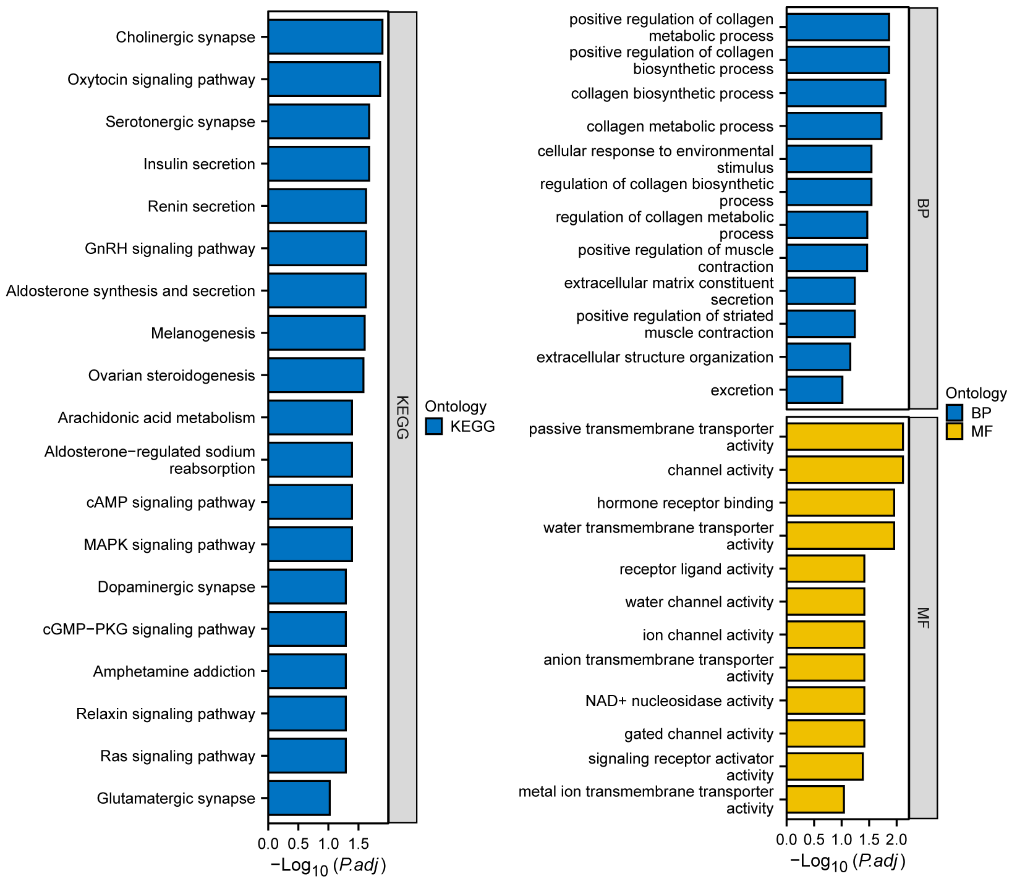
**

**Fig. 2.** Enrichment of other pathways caused by R11 interfering with bladder cancer cells in RNA-seq.


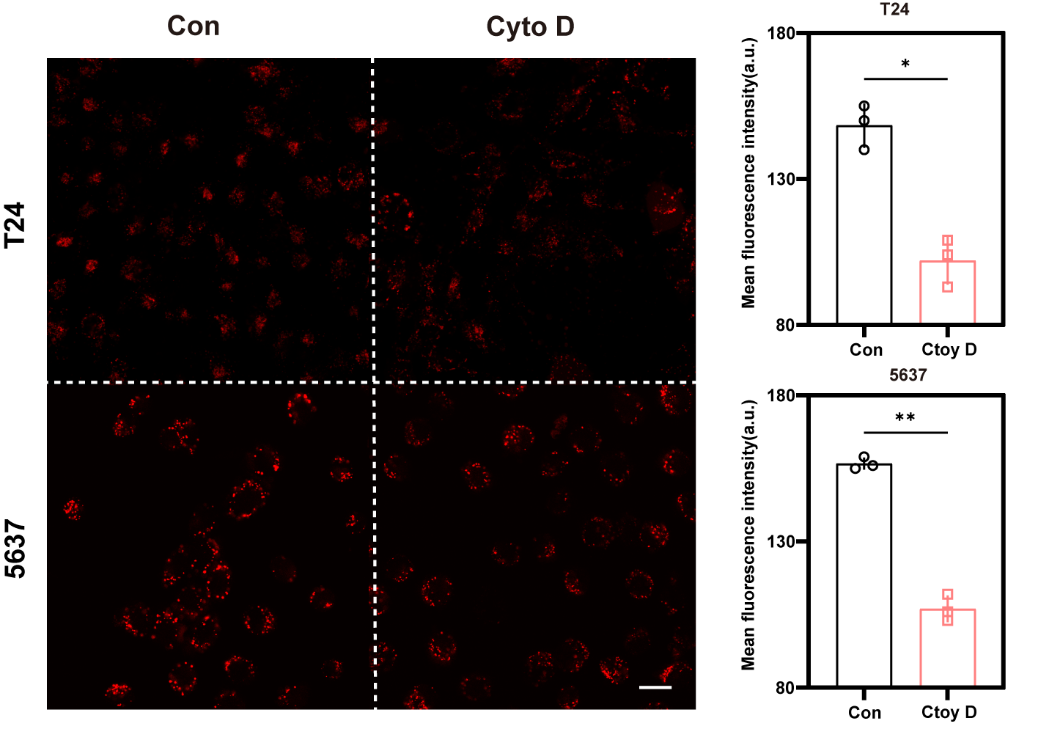


**Fig.** **3.** The inhibitory effect of Cyto D on the transmembrane transport of R11 Cyto D, illustrated by the CLSM analysis. R11 was observed from TARMA-fluorescence channel (Left insets). The fluorescence intensity of intracellular R11 (Right insets). **p*< 0.05, **p< 0.01.


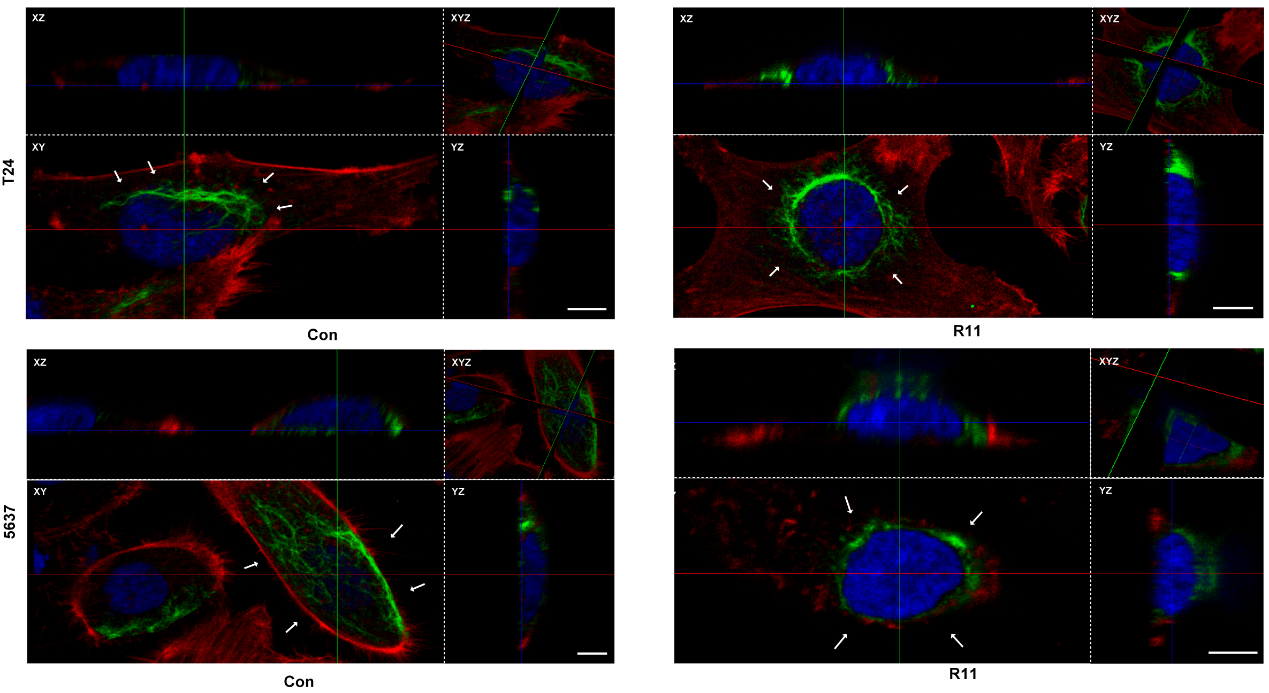


**Fig.** **4.** The representative super-resolution CLSM image of F-actin and vimentin in T24 and 5637 cell lines under 3D view; nucleus was observed via DAPI-fluorescence channel; F-actin was stained with the TRITC-phalloidin and observed via TRITC-fluorescence channel; vimentin was stained with the Alexa fluorescence 488 and observed via Alexa-488 fluorescence channel. R11 (5 μM), 24 h incubation; scale bar: 10 μm.


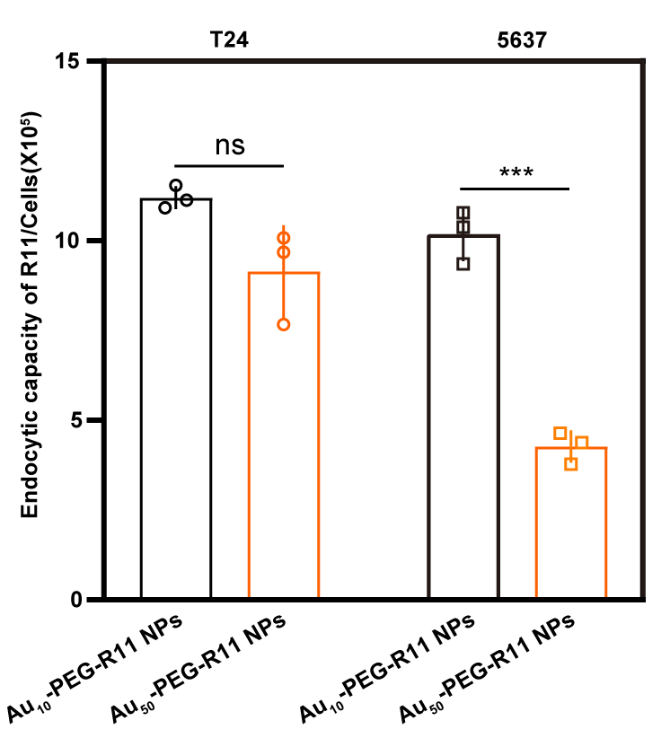


**Fig.** **5.** Cellular uptake of R11 assemblies of different size. R11 (10^-6^ μmol). **p* < 0.05.
