# Supplementary figures and images for: Polyarginine Peptide R11–Actin Interaction Induces a Domino Effect on Cytoskeleton Remodeling to Suppress Bladder Cancer Metastasis
Source: Research (Wash D C). 2026 Jan 29;9:1109. doi: 10.34133/research.1109 (PMC12852570; doi:10.34133/research.1109)

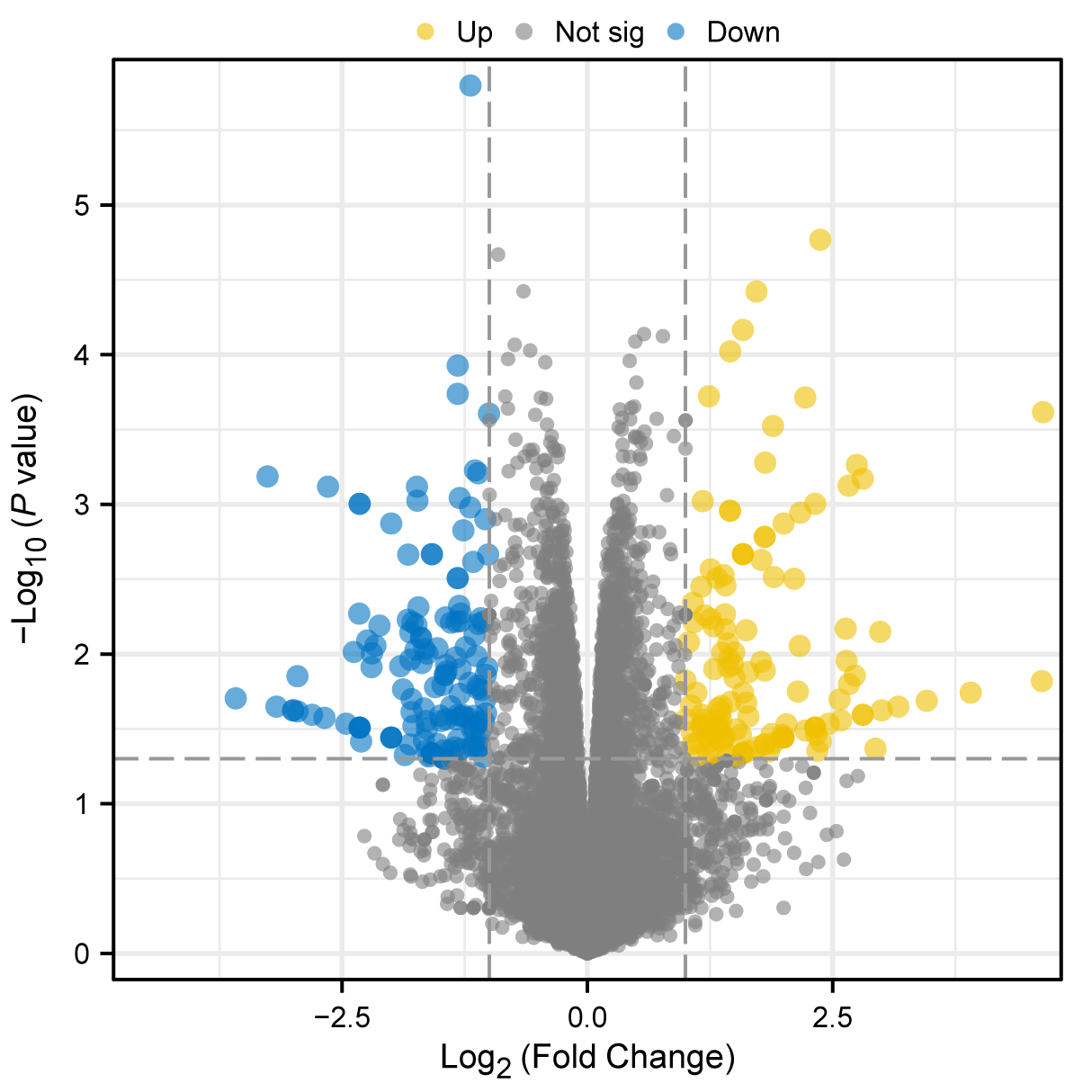

Supplement: Supplementary 1 — Figs. S1 to S5 [file research.1109.f1.zip › SI 1.tif]

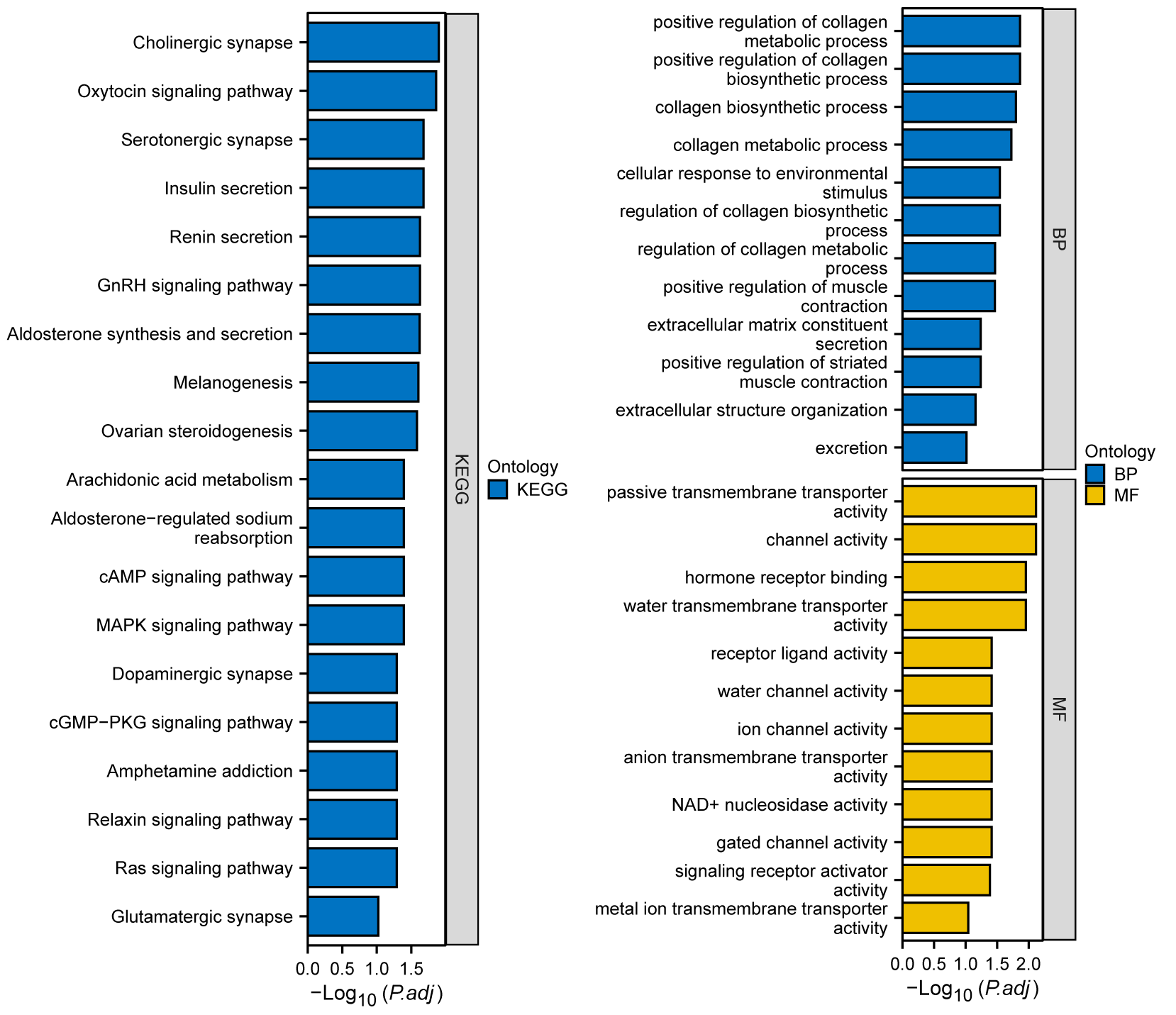

Supplement: Supplementary 1 — Figs. S1 to S5 [file research.1109.f1.zip › SI 2.tif]

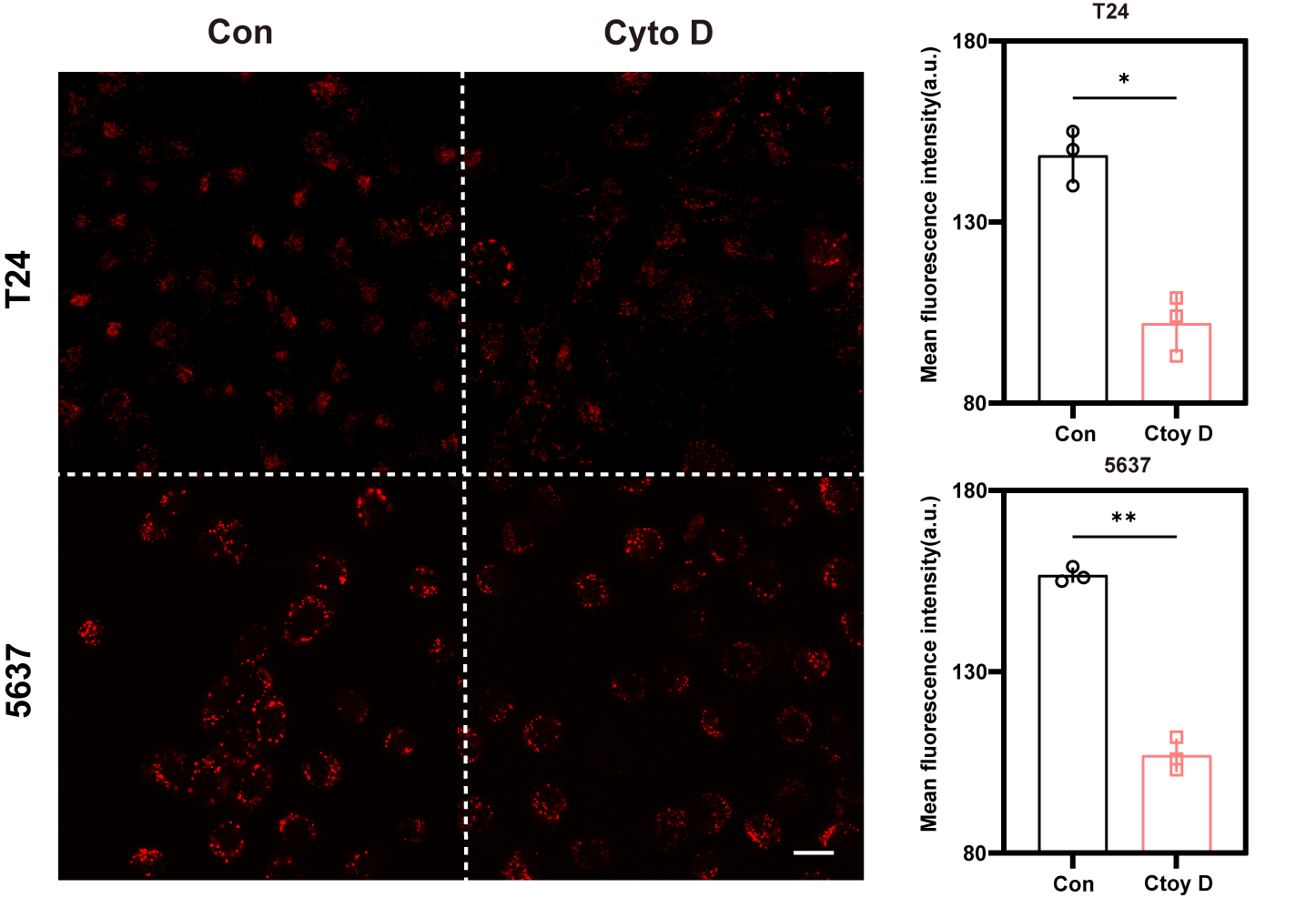

Supplement: Supplementary 1 — Figs. S1 to S5 [file research.1109.f1.zip › SI 3.tif]

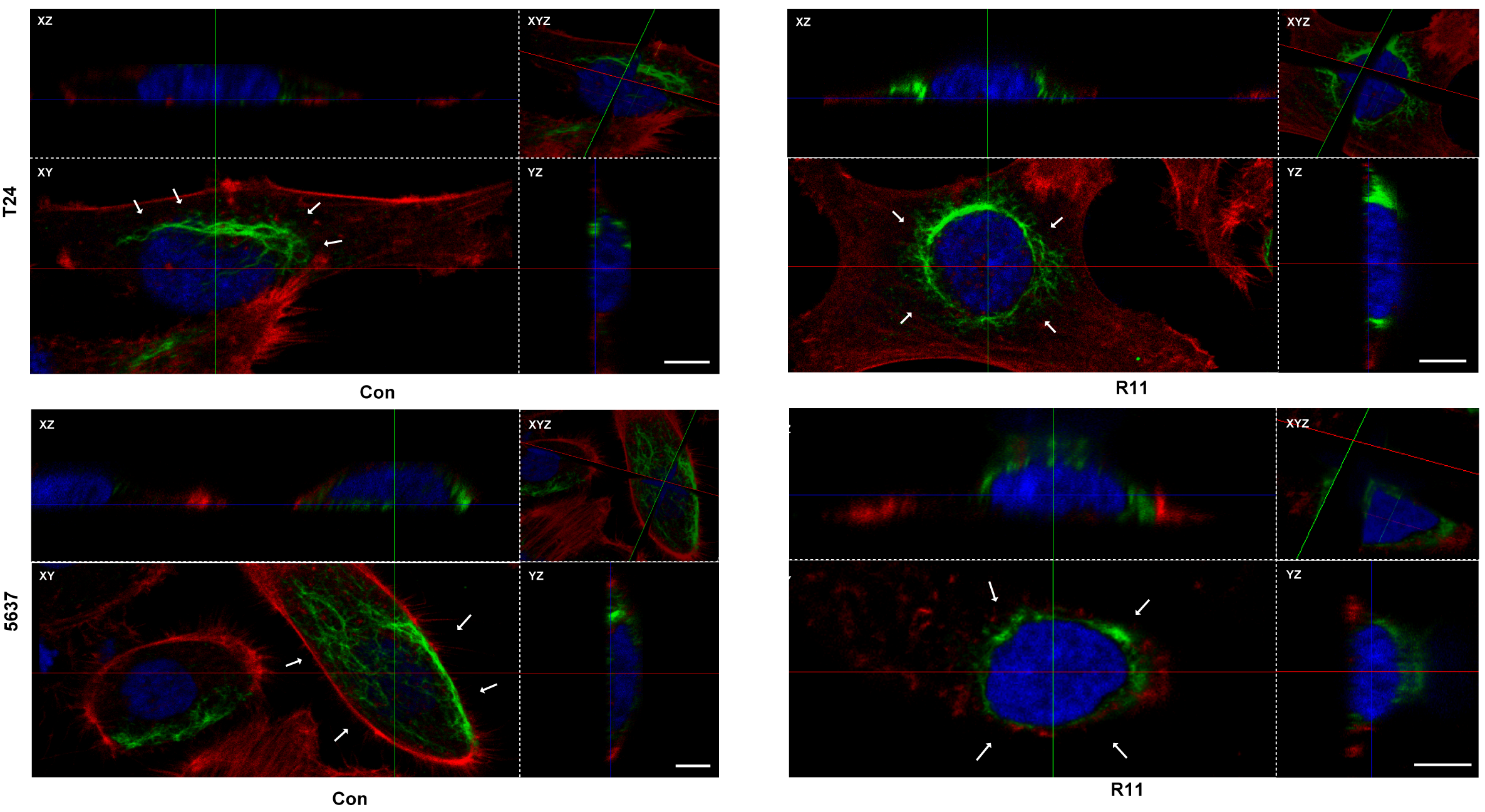

Supplement: Supplementary 1 — Figs. S1 to S5 [file research.1109.f1.zip › SI 4.tif]

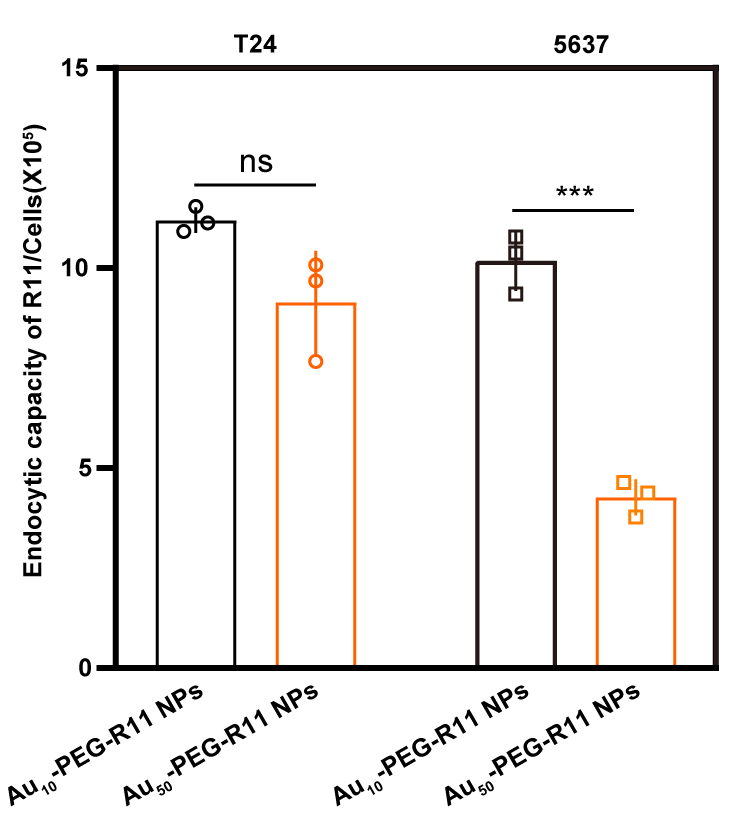

Supplement: Supplementary 1 — Figs. S1 to S5 [file research.1109.f1.zip › SI 5.tif]
